# Effectiveness of community-based interventions for PTSD among youth in low- and middle-income countries affected by humanitarian emergencies: A systematic review and meta-analysis

**DOI:** 10.1371/journal.pmen.0000602

**Published:** 2026-04-24

**Authors:** Gabrielle Drake, M Claire Greene, Alyssa Murdoch

**Affiliations:** 1 Department of Applied Psychology, New York University, Steinhardt, New York, New York, United States of America; 2 Program on Forced Migration and Health, Mailman School of Public Health, Columbia University, New York, New York, United States of America; 3 Department of Counseling and Clinical Psychology, Teachers College, Columbia University, New York, New York, United States of America; National Psychological Association of Ukraine, UKRAINE

## Abstract

Humanitarian emergencies expose millions of young people to adversity each year, with particularly severe consequences in low-and middle-income countries (LMICs) where mental health resources are scarce. In these contexts, community-based interventions (CBIs) can fill treatment gaps by leveraging local strengths to address mental health symptoms and sustainably foster resilience. Although these interventions demonstrate therapeutic potential, evidence regarding their effectiveness in reducing youth Post-Traumatic Stress Disorder (PTSD) symptoms remains mixed, particularly in LMICs affected by humanitarian emergencies. To examine the efficacy of CBIs for this population, a systematic review of five databases (Cochrane, Embase, PsycINFO, PubMed, and Scopus) was completed. Of the 1,687 records screened, 22 records met the inclusion criteria, 21 of which were included in analyses. Findings suggest a statistically significant, moderate effect of CBIs in reducing symptoms of PTSD in children and adolescents (g = -0.5). Further, exploratory moderator analyses suggests that incorporating individual sessions and culturally adapting interventions may enhance treatment outcomes. However, substantial heterogeneity across studies indicate that study effects vary widely. The findings from this review suggest that community-driven approaches are viable treatment options for traumatized youth in low-resource settings, offering valuable insights into the effectiveness of CBIs in high-risk contexts.

## Introduction

Humanitarian crises are a significant factor influencing youth outcomes worldwide. In 2025, UNICEF estimated that 213 million children across 146 territories required humanitarian assistance [1,2]. These crises pose substantial threats to the well-being of children and adolescents, including socioeconomic instability, displacement, family separation, heightened vulnerability to violence, and increased exposure to potentially traumatic events [3–5]. Almost all aspects of health are affected by humanitarian crises, increasing vulnerability to infectious disease, injury, physical trauma, malnutrition, and noncommunicable concerns, including mental health disorders [6]. Stressors in humanitarian contexts are more often chronic than acute, persisting in ways that create long-term psychological and physiological consequences for those affected [7]. In addition to heightened exposure to potentially traumatic events, populations in humanitarian settings may also face stigma surrounding mental health, lack of awareness of intervention options, and cultural insensitivity in treatment, all of which act as barriers to mental healthcare access [6,8].

The lack of safety associated with humanitarian emergencies has profound implications for the mental health and developmental trajectories of children and adolescents [9,10]. Of the approximately 225 million children and adolescents globally with a diagnosable mental health disorder, 197 million (88%) live in LMICs [11]. In LMICs, mental health disorders exacerbate developmental challenges, impair cognitive and social functioning, and increase the likelihood of school dropout [12]. The prevalence and risk of trauma-related mental health disorders are heightened in humanitarian settings due to the extreme and destabilizing conditions that characterize complex emergencies [13, 14]. This is especially true in LMICs, where limited resources compound this risk. In LMICs, PTSD prevalence is three times higher (prevalence of 15.3%) among those exposed to violent conflict or war [15]. A meta-analysis of the impact of war and forced displacement on youth mental health showed similar rates, with 22.7% of young refugees and asylum seekers meeting criteria for PTSD [16]. This is over four times the 5% general lifetime prevalence of PTSD in school-age youth in the United States [17]. Despite these increased rates, only 1 in 4 people with PTSD in LMICs report seeking any form of treatment [18]. Moreover, rates in LMICs are underestimated, as underreporting and barriers to diagnosis may obscure the true scope.

The impacts of PTSD are expansive across many aspects of life, including relationships, health, and overall sense of safety [19, 20], making treatment integral to long-term well-being. Young people who have experienced potentially traumatic events are at an increased risk of adverse mental health outcomes, including depression, bipolar disorder, suicide, and substance abuse [21–24] as well as physical health consequences, such as poor self-rated health, functional limitations, diabetes, and heart attack [25–27]. Children and adolescents diagnosed with PTSD face roadblocks in achieving healthy emotional, intellectual, and social development, compromising their cognitive functioning, ability to self-regulate, and capacity for resilience in response to additional trauma [10,28–35]. The long-term effects of early traumatic experiences are mediated by protective factors, including supportive relationships, stable environment, and emotion-regulation skills, emphasizing the importance of early intervention [32,36,37]. Given the unique developmental needs and vulnerability of traumatized youth and the pervasive nature of trauma in humanitarian settings, it is critical to examine treatment options that are feasible, effective, and sustainable in humanitarian contexts.

Implemented outside of traditional healthcare settings, CBIs refer to mental health and psychosocial support (MHPSS) interventions that operate across educational, familial, and social levels [38]. CBIs often involve organizations such as schools, community agencies, and social networks to engage participants as active contributors to these interventions, rather than passive recipients [39,40]. These interventions can provide an integrated and flexible approach to addressing and preventing the development of psychological disorders, fostering a sense of safety, and allowing for greater community connectedness [41]. Widely implemented in low-resource settings, CBIs have a demonstrated ability to accommodate limited resource availability and enhance untapped protective factors (i.e., social connections, access to education, emotional regulation) to improve psychosocial well-being [42,43]. As such, CBIs are a practical and effective approach that are uniquely positioned to leverage strengths already present in communities [44–47].

CBIs have demonstrated effectiveness in reducing symptoms of anxiety, depression, suicidality, and behavioral disorders among child and adolescent populations, including those living in LMICs affected by humanitarian emergencies [48–50]. Even in cases of severe mental illness, CBIs have been associated with lower rehospitalization rates [51]. By engaging the social-ecological systems surrounding children and adolescents, CBIs address psychosocial distress while accommodating external factors, such as family dynamics, peer influences, community resources, socioeconomic conditions, and cultural norms [52]. Despite the demonstrated efficacy of CBIs on the whole, evidence supporting their effectiveness in addressing youth PTSD symptoms, particularly in LMICs affected by humanitarian emergencies, are variable [28,30,53–61].

In an effort to bridge this gap in the literature, this review seeks to evaluate the effectiveness of CBIs in treating PTSD and related symptoms among young people in contexts where exposure to potentially traumatic events is both prevalent and severe. Further, this review aims to add nuance to any determined efficacy, examining which approaches are most effective and exploring what key facets may contribute to this effectiveness. Building upon existing literature examining how CBIs operate in settings of extreme adversity and resource limitations, this review will provide insight into the value of further adapting and resourcing CBIs for high-risk settings, particularly addressing PTSD as it affects children and adolescents. Given the variability in existing findings and the limited syntheses focused specifically on PTSD outcomes in humanitarian LMICs, this review aims to estimate the overall effect of CBIs on youth PTSD symptoms and explore intervention and study characteristics that may potentially be associated with variation in effects.

## Methods

**Fig 1:**
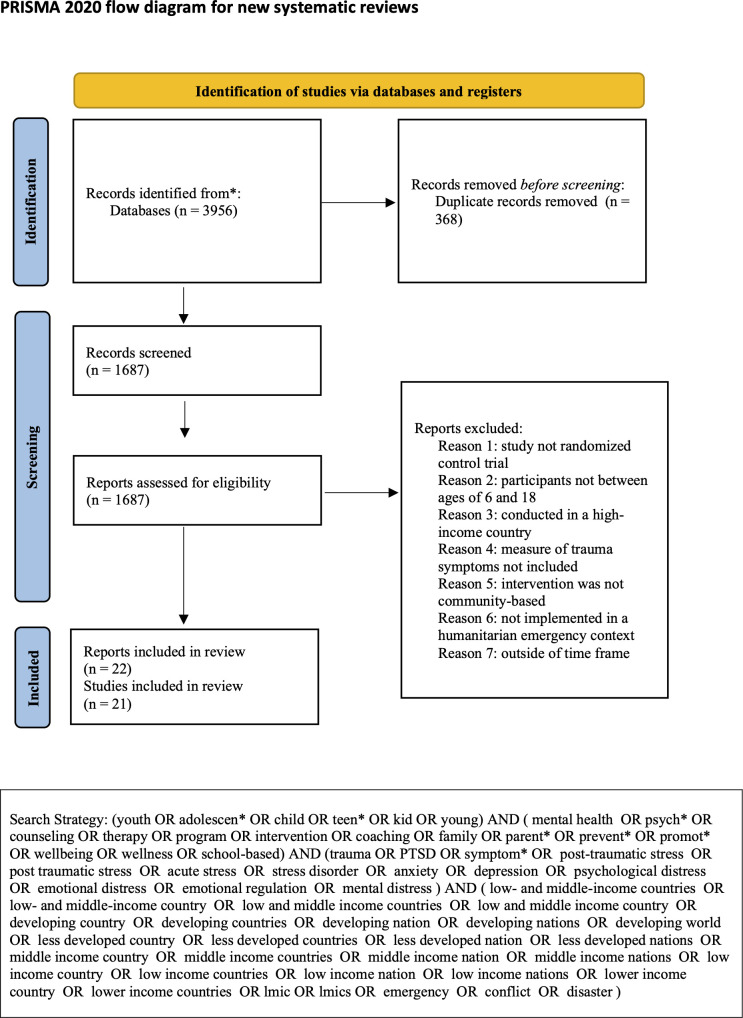
PRISMA Flow Chart.

The review was registered on the Open Science Framework (OSF). All data, supplementary materials, protocols, and analytic methods are publicly available at: https://osf.io/wygpv/overview?view_only=5f78fe924bbe4006a0eb0448b626b7a1. The protocol was developed and followed from project outset but registered retrospectively with OSF following review completion (January 2026). This systematic review followed PRISMA guidelines [62, 63]. We included studies published from January 2010 to January 2025 in our search. We searched five academic bibliographic databases, including Cochrane, Embase, PubMed, PsycInfo, and Scopus, using the search terms displayed in Fig 1. We did not limit results by including “randomized controlled trial” or “RCT” in the search string as to gain a comprehensive picture of the literature, including reviews, meta-analyses, and randomized controlled trials (RCTs). However, only RCTs were included in the review itself. We manually searched the citations of relevant systematic reviews on Google Scholar to ensure relevant studies were not missed. Title/abstract and full-text screening were conducted by a single reviewer (GD) under close supervision by a senior researcher to ensure that no relevant studies were missed. Data extraction was independently performed by two reviewers (GD and AM) following a pre-specified protocol, with the senior researcher providing oversight throughout the process to ensure accuracy and completeness. Inter-rater reliability for data extraction was high (97.8%), with discrepancies identified through comparison and resolved through discussion to reach consensus.

The research question followed PICOTS format [64]. The population was defined as individuals between the ages of 6-to-18 years, with the outcome of interest being symptoms associated with PTSD. During data extraction, all included studies had participants who were confirmed to fall within the specified age range of 6–18 years, with none of the studies including participants outside this range. Many studies of community-based and humanitarian interventions in LMICs define “youth” using broad age ranges (e.g., 10-to-24 or 10-to-19 years) rather than narrowly targeting specific subgroups. This is often done to ensure maximal access and to avoid excluding vulnerable young people simply because they fall outside strict age-based cutoffs [65,66]. These practices support our decision to use the 6-to-18-year age range to define youth in order to be inclusive of a wider range of studies and reflect what has been implemented in the field [67].

CBIs were defined as mental health interventions delivered outside of formal healthcare systems and in community settings. These interventions are aimed toward “provid[ing] or promot[ing] psychosocial well-being and prevent[ing] or treat[ing] mental health disorders” [68]. CBIs were defined broadly to capture the full range of interventions targeting traumatized children, including those implemented within school settings. Although schools may not be considered a purely community-based environment, they often function as central community hubs in low- and middle-income contexts and serve as key platforms for delivering psychosocial support. This same grouping has also been done in other reviews of mental health interventions [48,69]. Therefore, school-based interventions were included in the definition of CBIs to ensure a comprehensive synthesis of relevant evidence and to avoid excluding closely related initiatives. The outcome measure of trauma symptoms was defined as the presence of symptoms consistent with DSM-defined PTSD [19], following exposure to a specified potentially traumatic event or series of events. As reported by the study authors, studies included in this review explicitly focused on trauma-related symptoms.

The setting was LMICs affected by humanitarian emergencies. We defined LMIC settings using the World Bank country classification for the study period [70]. The full list of countries used to identify LMIC studies can be found on OSF. Where possible, searches were filtered by country names/regions. Otherwise LMIC status was applied at title/abstract or full-text screening. Humanitarian emergencies were defined as disasters or crises that overwhelm a community’s capacity to respond [71]. Studies were conducted in one of three humanitarian contexts: (a) conflict- or war-affected settings, (b) displacement settings, or (c) natural disaster emergency settings. These classifications were guided by typologies established in previous reviews [72]. In sum, studies were included if they were (1) randomized control trials (RCTs) implementing a (2) community-based intervention (CBI) within a (3) low- and middle-income country (LMIC) experiencing a (4) humanitarian emergency, specifically focusing on the population of (5) youth (defined as individuals ages 6–18), and addressing (6) trauma symptoms as a main outcome.

When multiple follow-up time points were reported, the immediate post-intervention assessment was selected to maximize comparability across studies. When multiple intervention arms were present, the arm most consistent with the definition of community-based interventions was included. When multiple publications reported the same trial, only the primary outcome dataset was included in the meta-analysis to avoid double-counting. Only one effect size per study was included in the primary analysis. Data extraction aimed to collect information surrounding participant and intervention characteristics, implementation, and study outcomes. Reviewers used a prespecified protocol to guide the data extraction process. The extracted data was used in subsequent qualitative and quantitative analyses. We conducted a narrative synthesis of the study characteristics, classifying interventions into the broad categories of: Cognitive Behavioral Interventions, Narrative Exposure and Related Processing Interventions, Psychosocial Programs, and Body-Mind Interventions (Table 1). While there was occasional crossover in the strategies (i.e., a psychosocial intervention utilizing cognitive behavioral techniques), interventions were grouped based upon their primary focus, stated goals, and overarching orientation.

**Table 1:** Description of Intervention Characteristics.

Type	Description
Cognitive Behavioral	These interventions utilize evidence-based, cognitive behavioral strategies, including psychoeducation, normalization of stress reactions, cognitive coping skills training, and behavioral techniques to alleviate trauma-related symptoms. Within this context, many of these interventions were trauma-informed. Most are manualized, offering structured opportunities for cognitive restructuring, challenging of maladaptive beliefs, and development of self-regulation skills. Supplemental individual sessions may be included to address sensitive content. Added caregiver sessions serve to enhance parent-child communication, foster supportive interactions, and educate families about PTSD symptomatology [73,74].
Narrative Exposure and Related Processing	Supporting the construction of a trauma narrative to process traumatic experiences, these interventions emphasize the expression of thoughts, feelings, and the discussion of the impact of adversity. The focus is on memory recall, particularly the sensory aspects of traumatic events, and how these memories are used for coping. The goal is to contextualize and integrate trauma into broader life stories, lessening the emotional impact, symptoms, and weight that it carries. Activities include writing, verbal discussion of trauma, and creative expression (i.e., drawing) as a means of exposure and narrative construction [75].
Psychosocial	Psychosocial interventions are focused on supporting youth adjustment after traumatic experiences, facilitating resilience through coping strategies, prosocial behavior, group cohesion, and hope. This is accomplished by establishing safe spaces, providing social support, and structured group-based activities for self-expression. Many of these interventions are classroom-based, implemented by paraprofessionals trained to promote psychosocial well-being. Activities include guided play, psychoeducation, stress management, creative expression, and problem solving [76].
Body-Mind	These approaches are geared toward improving mindfulness and self-awareness, using these skills as a means through which to address the physiological symptoms of post-traumatic stress, promoting physiological self-regulation and coping skills. This is accomplished by activities to integrate the mind and body, such as deep breathing, movement, and guided imagery [77].

Prespecified analyses included random-effects meta-analysis (Hedges’ g), meta-regression and subgroup analyses examining intervention characteristics, and study quality assessment using the Downs and Black Checklist (DBC), as documented in the protocol. Post hoc analyses were conducted to evaluate the influence of methodological and statistical assumptions on the pooled effect estimates. These included a series of sensitivity analyses (leave-one-out analyses, influence diagnostics, and assessment of potentially influential studies) as well as examining the impact of clustering using Intraclass Correlation Coefficients (ICCs). Pre- and post-intervention changes from baseline (T1) to post-intervention (T2) are reported for both the intervention and control groups in Table 3, with negative values indicating reductions in symptoms over time. We also calculated within-group standardized mean change effect sizes (Cohen’s d) using the pooled pre–post standard deviation. These descriptive pre–post changes are presented separately from the effect size estimates.

**Table 3:** Study Effects. Change in trauma symptoms.

			Study Context			Experimental Group			Control Group		
Study	DBC	Measure	Location	Emergency	Population	Group	T2-T1	d	Group	T2-T1	d
Ahmadi et al. [78]	23/28	CRIES- 13	Afghanistan	Conflict	100% F, 0% M; ages 12–18	m-WET (n = 34)	-4.88	-0.29	WLC (n = 38)	-3.28	-0.24
Ahmadi et al. [79]	23/28	CRIES- 13	Afghanistan	Conflict	100% F, 0% M; ages 11–19	METRA (n = 80)	-17.66	-0.89	TAU (n = 45)	-32.27	-0.19
Akhtar et al. [80]	20/28	CRIES- 13	Jordan	Displacement	50% F, 50% M; ages 10–14	EASE (n = 33)	-3.3	-0.18	ETAU (n = 26)	-4.64	-0.25
Barron, Abdallah, & Smith [81]	23/28	CRIES- 13	Palestine	Conflict	42% F, 58% M; ages 11–14	TRT (n = 90)	-5	-0.76	WLC (n = 50)	0.5	0.08
Barron, Abdallah, & Heltne [82]	23/28	CRIES- 13	Palestine	Conflict	60% F; 40% M; ages 11–15	TRT (n = 79)	-7.02	-0.88	WLC (n = 75)	-0.51	-0.07
Brown et al. [83]	19/28	CRIES- 13	Lebanon	Displacement	45% F, 55% M; ages 10–14	EASE (n = 35)	0.8	0.05	ETAU (n = 32)	-0.1	-0.01
Bryant et al. [84]	25/28	CRIES- 13	Jordan	Displacement	49.5% F, 50.5% M; ages 10–14	EASE (n = 185)	-5.84	-0.49	ETAU (n = 286)	-6.49	-0.51
Culver et al. [85]	20/28	UCLA PTSD RI	Haiti	Natural disaster	42% F 58% M; ages 7–17	Yoga (n = 15)	-9.67	-1.186	WLC (n = 15)	-1.43	-0.21
Dhital et al. [86]	22/28	CPSS	Nepal	Natural disaster	54% F; 46% M; ages 11–14	Training (n = 607)	0	0	ETAU (n = 615)	-0.6	-0.07
El-Khani et al. [87]	25/28	CRIES- 13	Lebanon	Displacement	not stated; ages 9–12	TRT + P (n = 41)	-18.77	-1.42	WLC (n = 40)	-3.43	-0.25
Fine et al. [88]	21/28	CPSS	Tanzania	Displacement	not stated; ages 10–14	EASE (n = 38)	-1.43	-0.192	ETAU (n = 44)	-0.82	-0.08
Getanda & Vostanis [89]	19/28	CRIES- 13	Kenya	Displacement	59% F, 41% M; ages 14–17	WfR (n = 25)	-27.7	-2.98	WLC (n = 25)	-2.4	-0.26
Jordans et al. [90]	23/28	CPSS	Nepal	Conflict and violence	50% F, 50% M; ages 11–14	Class -Based (n = 164)	-2.44	-0.47	WLC (n = 161)	-2.39	-0.41
Jordans et al. [91]	20/28	CRIES- 13	Lebanon	Displacement	49% F, 51% M; ages 10–14	EASE (n = 80)	0.6	0.03	ETAU (n = 118)	-1.5	-0.03
Lange- Nielsen et al. [92]	20/28	CRIES- 13	Palestine	Displacement	50% F, 50% M; ages 12–17	WfR (n = 66)	-3.39	-0.265	WLC (n = 58)	-2.87	-0.23
McMullen et al. [93]	25/28	UCLA PTSD RI	DRC	Conflict	0% F, 100% M; ages 13–17	TF-CBT (n = 25)	-26.5	-3.66	WLC (n = 25)	-2.5	-0.24
O’Callaghan et al. [94]	26/28	UCLA PTSD RI	DRC	Conflict	100% F, 0% M; ages 12–17	TF-CBT (n = 24)	-22.5	-2.18	WLC (n = 28)	2.64	0.21
O’Callaghan et al. [95]	25/28	UCLA PTSD RI	DRC	Conflict	50% F, 50% M; ages 8–17	TF-CBT (n = 26)	-26.23	-3.07	TAU (n = 24)	-4.72	-0.54
Panter-Brick et al. [96]	27/28	CRIES- 8	Jordan	Conflict	not stated; ages 12–18	TF-CBT (n = 269)	-0.49	-0.04	WLC (n = 194)	-0.99	-0.08
Qouta et al. [97]	25/28	CRIES 13	Palestine	Conflict	49% F, 51% M; ages 10–13	TRT (n = 242)	-6.85	-0.70	WLC (n = 240)	-0.35	-0.03
Tol et al. [98]	24/28	CPSS	Burundi	Conflict	48% F, 52% M; ages 8–17	Class-Based (n = 153)	-5.77	-0.58	WLC (n = 176)	-5	-0.57

Sample size reflects the number of participants whose data were included in the analysis (i.e., excluding those lost to follow-up).

For the primary analyses, pooled study effect size was calculated using Hedges’ g, a bias-corrected standardized mean difference, within a random-effects model using restricted maximum likelihood (REML). Effect sizes were computed based on between-group differences at post-intervention (T2) using the escalc function in R with measure = “SMDH.” That is, effect sizes were derived from post‑intervention scores in the intervention and control groups. This approach was used to maximize comparability across studies and to avoid reliance on change-score estimates, which were not consistently reported and would require assumptions about pre–post correlations that were not available. Negative values of g indicate lower PTSD symptom severity in the intervention group relative to the control group at post-intervention. All meta-analyses were conducted using R software (version 4.4.1) with the metafor package. Corresponding code can be found on OSF.

Between-study heterogeneity was evaluated using Cochran’s Q test (χ²), I² statistics, corresponding p-values, and was also visually inspected using forest plots. To explore potential sources of heterogeneity, meta-regression analyses were conducted by entering categorical study characteristics as moderators in separate mixed-effects models. These analyses allowed estimation of the proportion of between-study variance explained (R²) and comparison of subgroup effects relative to a reference category (model intercept). Given the relatively small number of included studies, moderator analyses were considered exploratory and intended to identify patterns rather than to support definitive conclusions.

Funnel plot asymmetry and small-study effects were assessed both visually and formally using Egger’s regression test to evaluate potential biases related to study size  [99].The Downs and Black Checklist (DBC) [100] was used to evaluate the quality of the studies’ methodologies, particularly assessing risk of bias. This quality assessment tool is ranked highly in its suitability for use in systematic reviews, demonstrating good psychometric properties, including internal consistency, test-retest reliability, inter-rater reliability, and criterion validity [101]. It was selected over other bias assessment tools because it provides a comprehensive evaluation of study quality across multiple domains while maintaining strong reliability and validity for diverse study designs. This approach was chosen to provide an objective and replicable quality assessment, allowing quantitative incorporation of study quality into sensitivity and moderator analyses, rather than relying solely on qualitative evaluations of bias. This checklist is rated out of 28, with a score of 28 indicating little to no bias. Two reviewers independently coded all studies using the DBC checklist. Observed agreement was 96.2%, with Cohen’s kappa (κ = 0.88) indicating substantial interrater reliability. A formal certainty assessment using the GRADE approach [102] was conducted for the primary outcome, drawing on quality ratings, heterogeneity, precision, and bias assessments; GRADE judgments and the corresponding Summary of Findings table are presented in S2 Table.

## Results

We identified 2,055 records, including articles searched on Google Scholar gathered from relevant systematic reviews. After removing duplicates, 1,687 records were screened for eligibility. Of these, 1,665 studies were excluded for not meeting the inclusion criteria, leaving 22 records that met the eligibility criteria and were included in the review. Of the 22 included articles, one represented a secondary analysis of an already included trial (Kangaslampi et al., [103], corresponding to Qouta et al., [97]) and was therefore excluded from the meta-analysis. It was retained for descriptive purposes but excluded from relevant descriptive counts to avoid double-counting (e.g., denominators of 21 rather than 22 where applicable).

The interventions used in the included studies were TF-CBT (Trauma-Focused Cognitive Behavioral Therapy; n = 3, 14.3%), TRT (Teaching Recovery Techniques; n = 3, 14.3%), TRT + P (Teaching Recovery Techniques + Parenting; n = 1, 4.8%), EASE (Early Adolescent Skills for Emotions; n = 5, 23.8%), m-WET (Modified Written Exposure Therapy; n = 1, 4.8%), METRA (Memory Training for Recovery – Adolescent; n = 1, 4.8%), WfR (Writing for Recovery; n = 2, 9.6%), Classroom-Based Interventions (n = 3, 14.3%), Advancing Adolescents (n = 1, 4.8%), and Hatha Yoga (n = 1, 4.8%) (Table 2).

**Table 2:** Intervention Characteristics.

CBI	Components	Study	Intervention Group Size	Dosage	Provider
Cognitive Behavioral					
TF-CBT	Manualized intervention incorporating psychoeducation, emotion regulation, coping, and processing to address maladaptive thoughts and behaviors.	McMullen et al. [93]	n = 25	15 group sessions (7 weeks; duration not stated) (3 caregiver & 2–4 individual sessions)	Professional
		O’Callaghan et al. [94]	n = 24	15 group sessions (5 weeks; 2 hours) (3 caregiver & 3 individual sessions)	Professional
		O’Callaghan et al., [95]	n = 25	9 group sessions (3 weeks; 1.5 hours) (2 caregiver & 1 individual sessions)	Teachers
TRT	Manualized intervention emphasizing coping and hope to normalize PTSD reactions and prevent further symptoms.	Barron, Abdallah, & Heltne [82]	n = 10	5 group sessions (5 weeks; 1.5 hours)	School Counselors
		Barron, Abdallah, & Smith [81]	n = 10	5 group sessions (5 weeks; duration not stated)	School Counselors
		Qouta et al. [97]	n = 15	15 group sessions (4 weeks; 2 hours)	Professional
TRT + P	TRT with added caregiver sessions focused on positive parent-child interactions.	El-Khani et al. [87]	n = 15	5 group sessions (5 weeks; 2 hours) (5 caregiver sessions)	Teachers
EASE	Trauma-informed utilization of psychoeducation and stress management to reduce internalizing symptoms.	Akhtar et al. [80]	n = 6–8	7 group sessions (7 weeks; 1.5 hours) (3 caregiver sessions)	Community Facilitator
		Fine et al. [88]	n = 12	7 group sessions (7 weeks; 1.5 hours) (3 caregiver sessions)	Community Facilitator
		Bryant et al. [84]	n = 10	7 group sessions (7 weeks; 1.5 hours) (3 caregiver sessions)	Community Facilitator
		Brown et al. [83]	n = 6–8	7 group sessions (7 weeks; 1.5 hours) (3 caregiver sessions)	Community Facilitator
		Jordans et al. [91]	n = 10	7 group sessions (7 weeks; 1.5 hours) (3 caregiver sessions)	Community Facilitator
Narrative Exposure and Related Processing					
m-WET	Writing and memory recall for trauma processing. Incorporates psychoeducation focused on coping.	Ahmadi et al. [78]	n = 5–8	5 sessions (5 days; 30 minutes)	Community Facilitator
METRA	Psychoeducation and memory recall to improve symptom-regulation. Paired with m-WET.	Ahmadi et al. [79]	n = 8	10 sessions (2 weeks; 1 hour)	Community Facilitator
WfR	Writing intervention targeting sensory aspects of trauma to promote emotional insight and symptom management.	Lange-Nielsen et al. [92]	Not stated	6 sessions (3 days; 15 minutes)	Community Facilitator
	WfR with parent psychoeducation.	Getanda & Vostanis [89]	Not stated	6 sessions (3 days; 15 minutes) (1 caregiver session)	Community Facilitator
Psychosocial					
Teacher Training	Training in crisis response and psychological well-being, exploring topics in security, identity, esteem, and hope.	Dhital et al. [86]	Class size (unspecified)	Incorporated into classroom for 6 months	Teachers
Classroom-Based	Multilevel classroom intervention incorporating themes of safety, awareness, coping, and reconnection.	Jordans et al. [90]	Class size (unspecified)	15 sessions (5 weeks; 1 hour)	Community Facilitator
Classroom-Based	Cognitive and creative approaches to decrease symptoms and strengthen protective factors.	Tol et al. [98]	n = 15	15 sessions (5 weeks; duration not stated)	Community Facilitator
Advancing Adolescents	Support adjustment by establishing safe spaces, social support, and structure through group activities.	Panter-Brick et al. [96]	n = 10–15	16 sessions (8 weeks; duration not stated)	Community Facilitator
Body Mind					
Hatha Yoga	Reintegration of mind-body processes through breathing exercises, yoga, and meditation.	Culver et al. [85]	n = 15	16 sessions (8 weeks; 45 minutes)	Research Assistants

The interventions were implemented across a variety of humanitarian emergencies, including contexts of conflict and community violence (i.e., armed conflict and war; n = 11, 52.4%), displacement (n = 8, 38.1%), and natural disaster (n = 2, 9.5%). In terms of geographic setting, as based upon WHO regional classifications [104], most studies were conducted in the Eastern Mediterranean (n = 12, 57.1%), specifically Afghanistan (n = 2, 9.5%), Jordan (n = 3, 14.3%), Lebanon (n = 3, 14.3%), and Palestine (n = 4, 19%). There were six studies conducted in sub-Saharan Africa (Burundi [n = 1, 4.8%]; Democratic Republic of Congo (DRC) [n = 3, 14.3%]; Kenya [n = 1, 4.8%]; Tanzania [n = 1, 4.8%]). The remaining three studies were set in the Americas (Haiti, n = 1, 4.8%) and South-East Asia (Nepal, n = 2, 9.5%).

Studies varied in their target age range, with some focusing broadly on young people ages 6–18 (n = 3, 14.3%), some focusing only on adolescents from ages 12 to 18 (n = 7, 33.3%), and some geared toward early adolescents ages 10–15 (n = 11, 52.4%). We did not conduct subgroup analysis for different ages due to the lack of distinction between developmentally established age groups in included studies. Definitions of these developmental stages are established in developmental literature, with middle childhood typically framed as approximately ages 6–11 years, early adolescence between 10–15 years, and adolescence extending into the later teenage years or until early twenties [105–107].

Most studies had an equal number of male and female participants (n = 14, 66.7%), with few focusing only on females (n = 3, 14.3%), and one focusing only on males (n = 1, 4.8%). Three studies did not specify their gender breakdown (n = 3, 14.3%). Gender was assumed to reflect the youth’s self-identified gender. Most studies were set in urban areas (n = 13, 61.9%) with few in rural areas (n = 5, 23.8%); three studies did not specify (n = 3, 14.3%). In terms of other population characteristics, seven studies focused on refugee youth (n = 7, 33.3%), one study focused on sexually abused youth (n = 1, 4.8%), and one study focused on an ethnic and religious minority group (n = 1, 4.8%). The few studies targeting marginalized groups reflect the paucity of research addressing the specific needs of vulnerable subpopulations within these contexts.

Most interventions were delivered in schools (n = 11, 52.4%). Other locations included non-governmental organization (NGO) sites within communities (n = 6, 28.6%) and refugee camps (n = 1, 4.8%), with the remaining intervention locations not specified or being held at various locations (n = 3, 14.3%). Dosage of the intervention varied. Few CBIs had 5 sessions (n = 3, 14.3%). Most were implemented between 5–10 sessions (n = 11, 52.4%). There were no studies implementing respective interventions between 10–15 sessions. Six interventions were implemented in 15 or more sessions (n = 6, 28.6%). One study did not state the number of sessions, rather that the intervention was disseminated over 6 months through the class curriculum (n = 1, 4.8%).

All interventions were delivered in group settings. Group sizes (for each intervention group, not total sample size) were mostly 15 participants or less (10 participants or less: n = 8, 38.1%; between 11 and 15 participants: n = 4, 19%). Fewer interventions had a group size greater than 16 participants, implementing interventions in class-sized groups (n = 4, 19%). Some studies did not explicitly state the number of participants per group nor indicate that they were implemented in a classroom-sized group (n = 5, 23.8%). Several studies incorporated a caregiver component (n = 10, 47.6%), with three of these also incorporating individual sessions (n = 3, 14.3%). The providers of these interventions were community facilitators (n = 12, 57.1%), professionals (i.e., psychologists; n = 3, 14.3%), teachers (n = 3, 14.3%), school counselors (n = 2, 9.5%), and research assistants (n = 1, 4.8%).

Across and within intervention categories, there were a variety of sub-components that differentiated them. Most followed a manualized protocol (n = 14, 66.7%), using a structured set of procedures and guidelines that standardize the delivery of the intervention. The remaining studies did not specify adherence to a specific protocol (n = 7, 33.3%). A creative component, such as writing, dancing, and physical movement, was incorporated into 9 studies (n = 9, 42.8%), geared toward promoting stress management and emotional regulation. Psychoeducation was incorporated into 13 studies (n = 13, 61.9%) to enhance understanding, engagement, and aid in the development of coping mechanisms. While psychoeducation is a cognitive behavioral technique [108], it was used across intervention categories. All interventions were reported to be culturally sensitive, demonstrating general awareness of and responsiveness to cultural factors (i.e., translation of materials to the participants’ native language and consultation with community stakeholders to ensure acceptability). However, only four studies explicitly described cultural adaptations, which involved intentional modifications to intervention content or delivery to align with the target culture (i.e., inclusion of culturally relevant examples or metaphors, adaptation of materials to local customs or beliefs, and involvement of community members in intervention design; n = 4, 19%).

The experimental groups were compared with various control conditions. Ten studies used a waitlist control (WLC; n = 10, 47.6%), five compared interventions to enhanced treatment as usual (ETAU; n = 5, 23.8%) and two used treatment as usual (TAU; n = 2, 9.5%). Three of these studies implemented more than one control group, including both an active and waitlist condition (n = 3, 14.3%). The non-active control was used as the comparator in analysis for consistency across studies. Studies measured the outcome variable of trauma symptoms through different means, largely conducting assessments at pre-intervention, post-intervention, and a follow-up evaluation. Studies without follow-up assessments beyond post-intervention (n = 8; 38.1%) [80–82,85,86,90,92,93] cited resource constraints, attrition, and the high-risk context as reasons for evaluating at baseline and post-intervention. For this reason, when comparing trauma symptoms before and after the intervention, we focused on scores from baseline (T1) and post-intervention (T2) as opposed to follow-up scores to maintain consistency across studies. Assessment tools include the Children’s Revised Impact of Event Scale-13 (CRIES-13; n = 12, 57.1%), Children’s Revised Impact of Event Scale-8 (CRIES-8; n = 1, 4.8%), UCLA PTSD Reaction Index (UCLA PTSD-RI; n = 4, 19%), and Child PTSD Symptom Scale (CPSS; n = 4, 19%).

Pre- and post-intervention changes from baseline (T1) to post-intervention (T2) are reported for both the intervention and control groups in Table 3, with negative values indicating reductions in symptoms from T1 to T2. We calculated within-group standardized mean change effect sizes (Cohen’s d) using the pooled pre–post standard deviation; negative values indicate a reduction in symptoms from T1 to T2 (Table 3). It should be noted that Barron, Abdallah, & Smith [81] presented T1 and T2 data through a visual model, indicating the mean and standard deviation as approximations. The effect size for the experimental group was stated in the article and thus is precise.

Quality assessment confirmed the robustness of the included studies. Based on the DBC, seven studies (n = 7, 33.3%) were rated as excellent (scores 25–28), 12 studies (n = 12, 57.1%) as good (scores 20–24), and two studies (n = 2, 9.5%) as fair (scores 15–19). The high proportion of good or excellent ratings (n = 19, 90.5%) indicates limited concerns regarding methodological quality, risk of bias, and validity concerns.

### Meta-analysis

**Fig 2:**
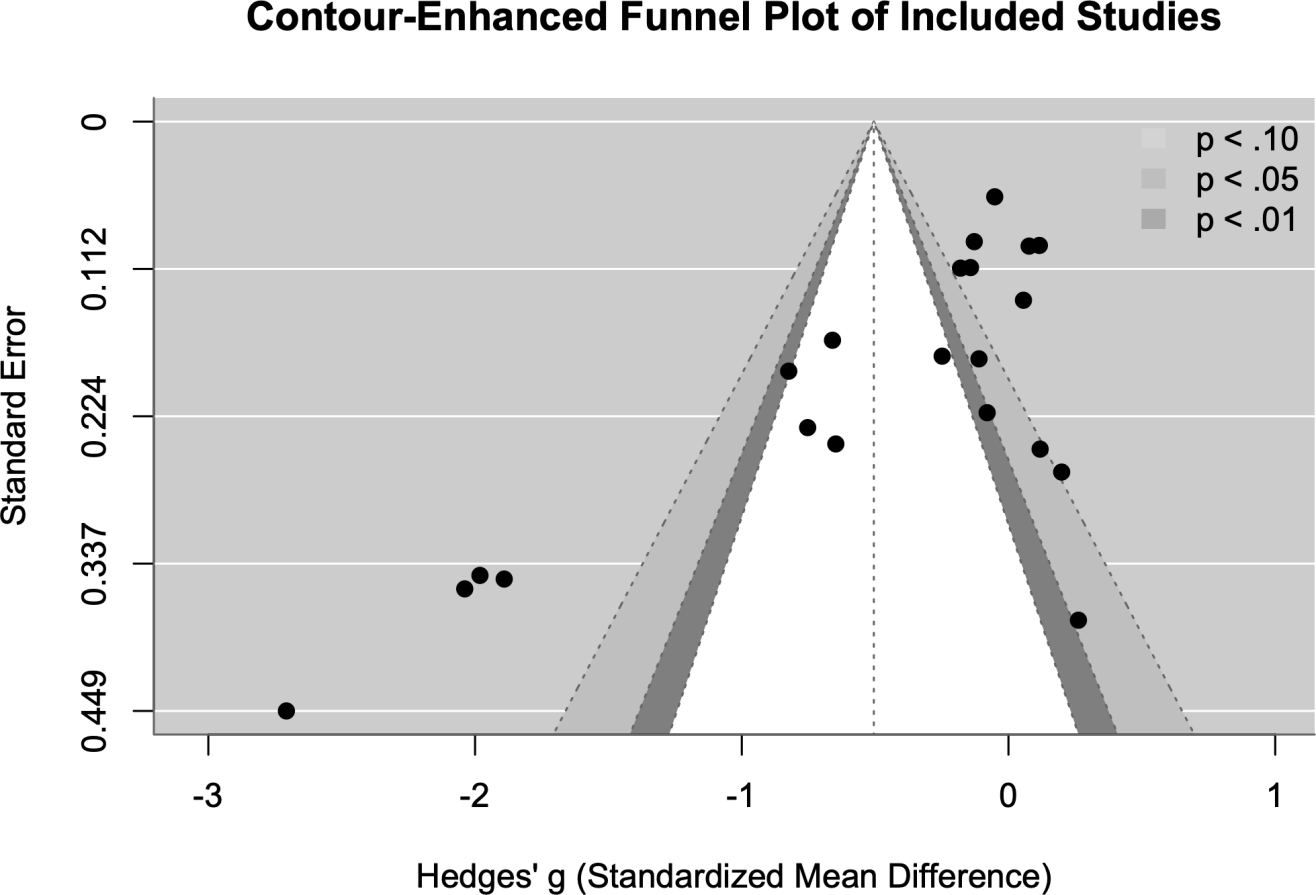
Funnel Plot. Assessing publication bias for post-intervention trauma symptoms.

**Fig 3:**
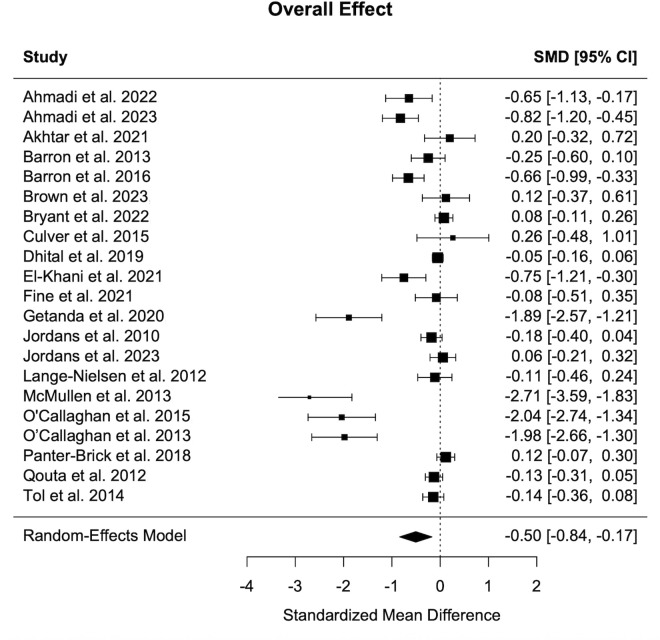
Forest Plot. Forest plot of pooled Hedges’ g for post-intervention trauma symptoms.

In the pooled analysis, the random-effects meta-analysis including 21 studies found that CBIs had a significant moderate effect on trauma related outcomes relative to controls (g = -0.5; 95% CI = [-0.84, -0.17]; p = 0.003). However, heterogeneity was substantial (τ² = 0.55; I² = 96.42%; Q (df = 20) = 170.0984, p < 0.001), suggesting true effect variation across studies. Funnel plot asymmetry (Fig 2) and Egger’s regression test confirmed statistically significant asymmetry (t = -3.78, df = 19, p = 0.0013), with a limit estimate of g = 0.28, and may suggest the presence of small-study effects. Consequently, we explored potential sources of this heterogeneity through moderator analyses, publication bias assessment, and sensitivity testing (Fig 3).

Post hoc sensitivity analyses indicated that the overall findings were robust across analytic assumptions. When varying assumed ICCs (0.01, 0.05, and 0.10) the pooled effect size remained identical (g = -0.51, SE = 0.17, p = 0.003), suggesting minimal impact of within-study clustering. Adjustment for baseline imbalance showed no association with effect size (β = 0.10, SE = 0.45, p = 0.818) and explained no heterogeneity (R² = 0%). Excluding the six studies with baseline imbalance (|g| > 0.20; k = 15 remaining) yielded minimal change in the effect size (g = -0.49, p = 0.023), confirming limited impact. Trim-and-fill analyses indicated no evidence of missing studies, with an adjusted estimate identical to the original observed effect (g = −0.50, SE = 0.17, p = 0.003). Leave-one-out analyses indicated robustness to the exclusion of any single study, as pooled estimates remained consistently negative and statistically significant (range: −0.54 to −0.41, all p < .01). Although a small number of studies (studies 16–18) showed relatively large residuals, none met formal criteria for influence based on Cook’s distance or DFFITS, and no studies were classified as influential. The model remained unchanged following influence assessments (g = −0.50, 95% CI [−0.84, −0.17], p = 0.003).

Heterogeneity remained high across all analyses (I² ≈ 96%), indicating substantial between-study variability that was not attributable to any single study. To identify sources of heterogeneity and features that moderated effect size, we ran a series of mixed-effects meta-regression analyses (Table 4).

**Table 4:** Moderator Analysis. Examining specific features as sources of heterogeneity in Hedges’ g for post-intervention trauma symptoms.

Moderator		Hedges’ g	Std Error (SE)	z-value	p-value
Intervention Type	Cognitive Behavioral	-0.9237	0.8813	-1.0481	0.2946
	Narrative	-0.9473	0.9183	-1.0315	0.3023
	Psychosocial	-0.3265	0.9298	-0.3511	0.7255
	Body-Mind (ref)	0.2623	0.8477	0.3094	0.757
Emergency	Natural Disaster	0.86406	0.5925	1.4581	0.1448
	Displacement	0.5049	0.3545	1.4240	0.1544
	Conflict (ref)	-0.7769	0.2304	-3.3727	0.0007 ***
Provider	Teacher	-0.6188	0.4731	-1.3079	0.1909
	School Counselor	-0.2008	0.5482	-0.3664	0.7141
	Professional	-1.2019	0.4877	-2.4643	0.0137 *
	Research Assistant	0.5166	0.8196	0.6303	0.5285
	Community Facilitator (ref)	-0.2543	0.2088	-1.2177	0.2233
Control Type	Enhanced Treatment as Usual	0.798	0.3846	2.0752	0.0380 *
	Treatment as Usual	0.2996	0.5466	0.5482	0.5836
	Waitlist (ref)	-0.7252	0.2002	-3.6223	0.0003 ***
**Subgroup Analysis**					
Individual Sessions	Yes	-1.9625	0.31863	-6.1603	<.0001 ***
	No	-0.2402	0.0965	-2.4891	0.0128 *
Caregiver Sessions	Yes	-0.5415	0.3381	-1.6015	0.1093
	No	-0.2814	0.2162	-1.3015	0.1931
School Setting	Yes	-0.2935	0.3454	-0.8498	0.3954
	No	-0.3536	0.2489	-1.4204	0.1555
Manualized	Yes	-0.3678	0.3606	-1.0199	0.3078
	No	-0.2620	0.2932	-0.8935	0.3716
Creative	Yes	0.2293	0.3512	0.6529	0.5138
	No	-0.6064	0.2314	-2.6200	0.0088 **
Psychoeducation	Yes	0.0339	0.3620	0.0937	0.9253
	No	-0.5286	0.2856	-1.8511	0.0642
Culturally Adapted	Yes	-1.2903	0.3650	-3.5347	0.0004 ***
	No	-0.2649	0.1498	-1.7676	0.0771

Significance codes: 0 ‘***’ 0.001 ‘**’ 0.01 ‘*’ 0.05 ‘’ 0.1 ‘’ 1.

Confidence Level 95%.

*(ref: reference group).*

### Moderator analysis

The multivariable model including intervention characteristics was statistically significant, QM (12) = 140.11, p < 0.001, and explained a substantial proportion of between-study variance (R² = 98.7%). Residual heterogeneity was low and non-significant (τ² = 0.007, QE (8) = 9.34, p = 0.315), suggesting that these moderators collectively accounted for much of the variability in effect sizes. Meta-regression models were used to examine whether each moderator accounted for variation across studies by estimating pooled effect sizes within each category. All moderators were included simultaneously to account for potential confounding between intervention characteristics. These findings should be interpreted cautiously. Given the small number of studies and potential collinearity among predictors, moderator analyses are underpowered and susceptible to Type I error. As such, these results are exploratory and hypothesis-generating rather than confirmatory.

*Intervention Type:* Tests of moderators indicated that intervention type was not a statistically significant moderator (QM (df = 3) = 2.83, p = .42), indicating no evidence that effect sizes differed across intervention categories. Subgroup estimates are presented in Table 4.
*Emergency:* Type of humanitarian emergency was not a statistically significant moderator (QM (df = 2) = 3.32, p = 0.19). Although some subgroup estimates reached statistical significance (conflict and community violence; g = -0.78, p = 0.0007) the overall test indicates insufficient evidence that intervention effects differ by setting (Table 4).
*Provider:* Provider type was not a statistically significant moderator (QM (df = 4) = 7.80, p = .09), suggesting no clear evidence that provider delivering the intervention explains variation in effect sizes (Table 4).
*Control Type:* Control conditions were categorized as waitlist (no active intervention), treatment-as-usual (TAU), or enhanced treatment-as-usual (ETAU) to examine whether differences in comparator conditions, and potentially the mere presence of psychosocial contact, were associated with variability in effect sizes. The control condition was not a statistically significant moderator (QM (df = 3) = 4.33, p = .115). Although some subgroup estimates reached statistical significance (waitlist; g = -0.73, p = 0.0003) the overall test indicates insufficient evidence that intervention effects differ by control group (Table 4)
*Additional sessions:* The inclusion of individual sessions was a statistically significant moderator of treatment effects, QM (df = 1) = 37.95, p < 0.0001, with interventions including individual sessions associated with larger effect sizes (g = -1.96, p < 0.0001). However, because all interventions that included individual sessions also incorporated caregiver components, these effects cannot be disentangled and may reflect overlapping or combined influences. Caregiver involvement (with no added individual child sessions) was not a statistically significant moderator (QM (df = 1) = 2.56, *p* = .11), suggesting no clear evidence that caregiver involvement in the intervention explains variation in effect sizes (Table 4).
*School Setting*: Intervention setting (school-based vs. non-school-based) was not a statistically significant moderator (QM (df = 1) = 0.72, p = .395), indicating no evidence that setting explains variability in effect sizes (Table 4).
*Intervention components:* Manualization (QM (df = 1) = 1.04, *p* = .31), psychoeducation (QM (df = 1) = 0.01, p = .93), and creative components (QM (df = 1) = 0.43, p = .51) were not statistically significant moderators, indicating no evidence that these features explain heterogeneity (Table 4). Cultural adaptation was statistically significant (QM (df = 1) = 12.49, p = 0.0004), with culturally adapted interventions being associated with larger reductions in trauma symptoms (g = -1.29, p = 0.0004).

Overall, in univariable meta-regressions, individual sessions (QM (1) = 37.95, p < 0.0001) and cultural adaptation (QM (1) = 12.49, p < 0.001) were found to be significant moderators of treatment effects. Interventions including individual sessions were associated with larger effects (g = -1.96, p < 0.0001) compared to those without. Culturally adapted interventions were associated with larger effects (g = −1.29, p = 0.0004) compared to non-adapted interventions. Other suspected moderators of treatment efficacy – including intervention type, emergency, provider, addition of caregiver sessions, control group, school setting, intervention manualization, creative components, and psychoeducation components – did not demonstrate significant moderation effects. However, the number of studies was relatively small (k = 21) compared to the number of predictors included in the multivariable model, increasing the risk of overfitting and unstable coefficient estimates; thus, moderator analyses are presented as exploratory and should be interpreted with caution.

## Discussion

Findings from this systematic review and meta-analysis indicate that CBIs are associated with a moderate reduction in trauma symptoms among youth in LMICs affected by humanitarian emergencies. Significant PTSD symptom reductions were found across interventions implemented in settings affected by conflict, displacement, and natural disaster, suggesting that CBIs may serve as a viable, scalable alternative to formal mental health services where specialized care is scarce. The findings that cultural adaptation and incorporation of individual sessions may moderate implementation outcomes highlights the importance of examining mechanisms of treatment efficacy in real-world settings.

Our exploratory finding that intervention type was not a statistically significant moderator of treatment efficacy may indicate that access to structured, evidence-based therapeutic contact, rather than adherence to a specific theoretical model, could play a meaningful role. Further, the lack of clear differences in effects by control group type raises the possibility that ETAU and TAU conditions may include components with some value, such as therapeutic contact, thereby potentially diminishing differences relative to the intervention. For practitioners operating in constrained humanitarian contexts, this pattern is encouraging, implying that offering a well‑delivered intervention grounded in evidence‑based principles can yield benefits in and of itself. However, the absence of statistically significant differences across modalities may also reflect limited power, heterogeneity in populations and delivery settings, and substantial overlap in common elements (e.g., exposure, emotion regulation, coping skills), rather than true equivalence. Future work should move beyond broad labels to identify specific processes, mechanisms, and shared components that drive change, testing elements that can be delivered efficiently in low‑resource environments. This is particularly true for Body-Mind interventions, of which only one was found in the study sample.

Addition of individual sessions, features largely embedded within cognitive-behavioral approaches, were associated with greater reductions in PTSD symptoms. Because all interventions that included individual sessions also incorporated caregiver participation, these effects cannot be disentangled and may reflect additive influences rather than the impact of individual contact alone. Although evidence comparing group-based and individual trauma interventions remains mixed [109], the present review suggests that incorporating at least some individualized attention within otherwise group-based CBIs may enhance treatment effects.

In this review, cultural adaptation was defined as the intentional integration of culturally salient materials and processes to tailor content and delivery to specific communities. This approach extends beyond basic cultural sensitivity, which aims to ensure an intervention is acceptable and appropriate (e.g., translation or general contextualization), by actively embedding local meanings, symbols, and practices into activities and framing [110,111]. Interventions which reported employing such adaptations were associated with larger reductions in PTSD symptoms. However, because this review defines cultural adaptation as a step beyond cultural sensitivity, the operational boundaries of this definition may not be standardized enough to fully capture the range of culturally incorporated material across studies. Nonetheless, this pattern suggests that cultural appropriateness should be considered a minimum standard for interventions, and that intentional, community-informed adaptation may confer additional benefits in engagement, acceptability, and perceived relevance. Because this review synthesized published trial data, youth narratives were not available for inclusion; future studies should incorporate participant voices to better understand perceived acceptability, mechanisms of change, and lived experience, which includes the perceived importance of culturally salient material. Clarifying the role of cultural adaptation is critical in humanitarian settings, where historical adversity, collective narratives, and local idioms of distress influence how youth interpret symptoms, engage with treatment, and move toward recovery.

## Strengths and limitations

This review’s focus on children and adolescents in LMICs affected by humanitarian emergencies addresses a critical gap in the evidence base, which is often centered on adults in crisis settings or youth in non-emergency contexts. Young people in humanitarian settings face layered vulnerabilities, including ongoing exposure to violence and loss, disrupted schooling, economic hardship, and limited access to formal care [112]. Trauma in youth is frequently complex and chronic, with symptoms that can be overlooked or misdiagnosed, underscoring the urgent need for sustainable, evidence-based interventions [113]. By synthesizing RCTs of CBIs specifically targeting PTSD symptoms in this population, the present review provides a more precise estimate of treatment effects than broader reviews focused on mixed mental health outcomes [47,114,115]. The findings are consistent with adult literature from LMIC humanitarian settings, demonstrating that community-based and task-shifted models can effectively reduce trauma-related and broader psychological symptoms [43,44,47,53,116], extending this evidence to youth PTSD outcomes specifically. Reviews of child-focused CBIs in LMICs that are not trauma-specific have reported mixed results [117], and similar variability has been observed for trauma-affected youth in high-income countries [118]. In contrast, the current review indicates that CBIs are associated with decreases in PTSD symptoms among youth in humanitarian LMIC settings. These findings suggest that community-driven designs and culturally responsive materials [119] may be particularly impactful when formal trauma services are limited, and when social support functions as a primary buffer against distress [46]. By focusing on trauma-specific outcomes in high-risk LMIC humanitarian contexts, this review complements prior work and highlights the potential of CBIs to leverage existing protective factors to support resilience and long-term recovery. Furthermore, by encompassing a range of program types implemented across diverse humanitarian crises, the review provides a comprehensive picture of how CBIs are currently used to support traumatized youth and identifies opportunities to strengthen intervention design and implementation.

This review has several important limitations. First, the protocol was registered retrospectively, after completion of the review. This reflects the study’s origin as a master’s thesis, where formal registration was not prioritized. Although prospective registration is preferable, all pre-specified analyses were conducted as documented in OSF, and any protocol amendments were transparently reported (S1 Table). Second, screening was conducted by a single reviewer, which may increase the risk of study selection bias. Third, heterogeneity was substantial, reflecting variation in intervention types, populations, and contexts. Thus, findings from analyses should be interpreted with caution. Fourth, many studies assessed outcomes only at post-intervention, limiting conclusions about durability of effects. Fifth, multivariable meta-regression analyses were likely underpowered, increasing the risk of Type I error and unstable estimates. In addition, overlap among moderator variables limited the ability to disentangle their independent effects; therefore, moderator findings should be interpreted cautiously and considered hypothesis-generating rather than confirmatory.

Limitations in the evidence base should also be noted. Several subgroups of interest, including younger children, religious and ethnic minorities, and youth experiencing intersecting marginalization, were underrepresented. For example, only a single trial focused on a religious and ethnic minority group [78], reflecting broader gaps in the evidence for the most vulnerable populations. The included studies were concentrated in the Eastern Mediterranean region (57.1%) and primarily conducted in conflict-affected settings (52.4%), which may limit the generalizability of findings to other humanitarian contexts. Although refugee populations were represented in some studies (33.3%), other marginalized groups were less frequently included. Importantly, socioeconomic status was inconsistently reported across studies, limiting our ability to assess disparities by income or class. Follow-up assessments were generally short (typically 3–6 months), and several studies reported only immediate post-intervention outcomes, limiting the ability to assess the durability of effects. Small sample sizes, attrition, and contextual challenges related to recruitment and retention may have reduced statistical power to detect nuanced moderator effects and introduced potential bias. Variation in control conditions may have contributed to the observed heterogeneity; however, restricting comparators to (enhanced) treatment-as-usual or waitlist controls allowed for more meaningful categorization and analysis. In addition, Body-Mind and other alternative community-based approaches were underrepresented due to the limited number of available studies. While this review focused on structured community-based interventions, a broader range of community-driven activities, such as recreational, creative, and social engagement programs, may also play an important role in supporting youth mental health in humanitarian settings. Future research should evaluate the effectiveness, feasibility, and cultural relevance of these approaches, particularly in LMICs where scalable and flexible interventions are critically needed. Despite these limitations, quality ratings indicated that the included RCTs met key methodological standards, supporting general confidence in the findings.

## Conclusion

Overall, this review reinforces that CBIs can substantially reduce PTSD symptoms among youth in humanitarian emergencies, with experimental groups consistently showing greater improvements than controls across diverse contexts. The findings underscore the potential of community‑delivered care to improve developmental trajectories for young people exposed to severe and ongoing adversity. Further, these findings highlight the importance of examining the role of individualized therapeutic contact and cultural adaptation in interventions and in influencing treatment outcomes. CBIs represent a promising and scalable approach, however, given substantial heterogeneity, further high-quality, contextually diverse trials with longer follow-up are needed to confirm these effects and confirm the role of these moderators beyond exploratory findings. Further, future trials would benefit from incorporating mixed methods approaches, including qualitative data, to capture youth perspectives and contextualize quantitative outcomes. For policymakers and implementing agencies, these results argue for sustained investment in CBIs that balance feasibility and scalability with attention to quality, supervision, and cultural fit. Strengthening local capacity to deliver, adapt, and refine such interventions through training, task‑sharing, and integration into existing systems offers a pathway toward more equitable care for trauma-exposed children and adolescents in LMICs affected by humanitarian crises.

## Supporting information

S1 Checklist PRISMA 2020 Checklist. Documents adherence to reporting guidelines across all sections of the systematic review and meta-analysis. From: Page MJ et al. (2021). The PRISMA 2020 statement: an updated guideline for reporting systematic reviews. BMJ, 372:n71. Used under the Creative Commons Attribution 4.0 International License (CC BY 4.0).
(DOCX)

S1 Text Registered Protocol. Details search strategy, eligibility criteria, data extraction plan, and analysis methods.
(DOCX)

S1 Table Protocol Amendments. Documents deviations between the original master’s thesis plan and actual conducted review, with rationale, timing, and impact assessments.
(DOCX)

S2 Table GRADE Summary of Findings. For the primary outcome comparing community-based interventions to control conditions.
(DOCX)

S1 Fig Regional Distribution. Pie and bar chart showing distribution of studies by geographic region.
(TIFF)
