# Supplementary material for: Effectiveness of community-based interventions for PTSD among youth in low- and middle-income countries affected by humanitarian emergencies: A systematic review and meta-analysis
Source: PLOS Ment Health. 2026 Apr 24;3(4):e0000602. doi: 10.1371/journal.pmen.0000602 (PMC13108866; doi:10.1371/journal.pmen.0000602)
Supplement: S1 Checklist — Documents adherence to reporting guidelines across all sections of the systematic review and meta-analysis. From: Page MJ et al. (2021). The PRISMA 2020 statement: an updated guideline for reporting systematic reviews. BMJ, 372:n71. Used under the Creative Commons Attribution 4.0 International License (CC BY 4.0). (DOCX) [file pmen.0000602.s001.docx]

| **Section and Topic** | **Item #** | **Checklist item** | **Location where item is reported** |
| --- | --- | --- | --- |
| **TITLE** | | |  |
| Title | 1 | Identify the report as a systematic review. | Title: “Effectiveness of community-based interventions for PTSD among youth in low- and middle-income countries affected by humanitarian emergencies: a systematic review and meta-analysis” (pg. 1). |
| **ABSTRACT** | | |  |
| Abstract | 2 | See the PRISMA 2020 for Abstracts checklist. | Abstract describes background, methods, results, and conclusions (pg. 2). |
| **INTRODUCTION** | | |  |
| Rationale | 3 | Describe the rationale for the review in the context of existing knowledge. | Introduction section, paragraphs 1–5, describing burden of PTSD among youth in LMIC humanitarian settings and prior CBI evidence (pgs. 2-5). |
| Objectives | 4 | Provide an explicit statement of the objective(s) or question(s) the review addresses. | At the end of the Introduction section is the statement: “this review seeks to evaluate the effectiveness of CBIs in treating PTSD… further, this review aims to add nuance to any determined efficacy…” (pg. 5). |
| **METHODS** | | |  |
| Eligibility criteria | 5 | Specify the inclusion and exclusion criteria for the review and how studies were grouped for the syntheses. | The Methods section includes paragraphs describing PICOTS, youth aged 6–18, RCTs, LMICs, humanitarian emergencies, CBIs, PTSD outcomes (pgs. 6-8). A summary paragraph of the inclusion criteria (beginning with: “In sum, studies were included if they were …”) can be found on page 8. |
| Information sources | 6 | Specify all databases, registers, websites, organisations, reference lists and other sources searched or consulted to identify studies. Specify the date when each source was last searched or consulted. | The Methods section includes the paragraph: “We included studies published from January 2010 to January 2025… We searched five academic bibliographic databases, including Cochrane, Embase, PubMed, PsycInfo, and Scopus… We manually searched the citations of relevant systematic reviews on Google Scholar…” (pg. 6). |
| Search strategy | 7 | Present the full search strategies for all databases, registers and websites, including any filters and limits used. | The Methods section includes a sentence “using the search terms displayed in Fig 1” (the PRISMA flow/search figure, which includes full strategies; pg. 6). |
| Selection process | 8 | Specify the methods used to decide whether a study met the inclusion criteria of the review, including how many reviewers screened each record and each report retrieved, whether they worked independently, and if applicable, details of automation tools used in the process. | The Method includes a description of title/abstract and full-text screening by a single reviewer (GD) under supervision of a senior researcher; no automation tools described (pg. 6) |
| Data collection process | 9 | Specify the methods used to collect data from reports, including how many reviewers collected data from each report, whether they worked independently, any processes for obtaining or confirming data from study investigators, and if applicable, details of automation tools used in the process. | The Methods section includes information about data extraction: “Data extraction was independently performed by two reviewers (GD and AM) following a pre-specified protocol… Inter-rater reliability for data extraction was high (97.8%), with discrepancies… resolved through discussion.” No automation tools or author contact (pg. 6). |
| Data items | 10a | List and define all outcomes for which data were sought. Specify whether all results that were compatible with each outcome domain in each study were sought (e.g. for all measures, time points, analyses), and if not, the methods used to decide which results to collect. | The Methods section includes a description of outcomes, defined as “symptoms associated with PTSD” with specific PTSD measures listed (CRIES-13, CRIES-8, UCLA PTSD-RI, CPSS), description that trauma symptoms were primary outcome, focusing on baseline (T1) to post-intervention (T2) scores (pg. 6). |
|  | 10b | List and define all other variables for which data were sought (e.g. participant and intervention characteristics, funding sources). Describe any assumptions made about any missing or unclear information. | The Methods section includes a description of data extraction “aimed to collect information surrounding participant characteristics, intervention implementation, and study outcomes,” resulting in a peripheral focus on detailed study characteristics (age, gender, setting, provider, dosage, group size, cultural adaptation, manualization, creative components, caregiver sessions, individual sessions, control conditions). No assumptions were made about missing/unclear information nor were described (pg. 8). |
| Study risk of bias assessment | 11 | Specify the methods used to assess risk of bias in the included studies, including details of the tool(s) used, how many reviewers assessed each study and whether they worked independently, and if applicable, details of automation tools used in the process. | The Methods section includes a description of risk assessment tools. “The Downs and Black Checklist (DBC) was used to evaluate the quality of the studies’ methodologies, particularly assessing risk of bias… Two reviewers independently coded all studies using the DBC checklist. Observed agreement was 96.2%, with Cohen's kappa… 0.88.” No automation tools described or used (pgs. 9-10). |
| Effect measures | 12 | Specify for each outcome the effect measure(s) (e.g. risk ratio, mean difference) used in the synthesis or presentation of results. | The Methods section includes a description of measures of effect size: “Each study’s effect size was computed as Hedges' g (g), the bias-corrected standardized mean difference, comparing post-intervention (T2) and baseline (T1) symptom scores in experimental and control groups. A negative Hedges’ g indicated symptom reduction from T1 to T2” (pgs. 8-9). |
| Synthesis methods | 13a | Describe the processes used to decide which studies were eligible for each synthesis (e.g. tabulating the study intervention characteristics and comparing against the planned groups for each synthesis (item #5)). | The Methods section details the inclusion of RCTs only and the necessity of meeting inclusion criteria included in the primary synthesis. Description of categorization in Methods and categorization of interventions in Table 1 (pgs. 6-10). |
|  | 13b | Describe any methods required to prepare the data for presentation or synthesis, such as handling of missing summary statistics, or data conversions. | The Results section describes PTSD scores converted into effect sizes for standardized comparison (pg. 18). Mention of Barron, Abdallah, & Smith trial where T1/T2 data approximated visually, effect size used from explicit text in the article. No further detail on imputation/other missing data handling is included (pg. 18). |
|  | 13c | Describe any methods used to tabulate or visually display results of individual studies and syntheses. | The Results section includes a description of forest plots (Fig 3), funnel plot (Fig 2) (found on page 20), and tables of intervention characteristics (Table 2; pg. 12-13), and study effects (Table 3) (pg. 18-19). |
|  | 13d | Describe any methods used to synthesize results and provide a rationale for the choice(s). If meta-analysis was performed, describe the model(s), method(s) to identify the presence and extent of statistical heterogeneity, and software package(s) used. | The Methods section describes “estimat[ion of] the pooled effect of CBIs on PTSD symptoms using a random-effects model” (pg. 8-9). Results describe heterogeneity statistics reported (τ², I², H²) and Cochran’s Q, with pooled Hedges’ g and 95% CI (pg. 20). The software package used was R (pg. 9). |
|  | 13e | Describe any methods used to explore possible causes of heterogeneity among study results (e.g. subgroup analysis, meta-regression). | The Methods/Results section includes a description of mixed-effects meta-regression analyses with moderators (intervention type, emergency type, provider, manualization, creative components, psychoeducation, cultural adaptation, individual sessions, caregiver sessions) (pg. 9). Table 4 includes results of the Moderator Analysis (pg. 20). |
|  | 13f | Describe any sensitivity analyses conducted to assess robustness of the synthesized results. | The Methods section describes how there was no explicit sensitivity analysis (e.g., exclusion by risk of bias, small-study effects) (pg. 9), only moderator analyses and publication bias visuals are reported (pgs. 9-10). |
| Reporting bias assessment | 14 | Describe any methods used to assess risk of bias due to missing results in a synthesis (arising from reporting biases). | The Methods/Results details how “visual assessments for publication bias were conducted using funnel plots and forest plots” (pg. 9). “Visual inspection of the funnel plot (Fig 2) indicated moderate asymmetry. However, the quality assessment confirmed the robustness” (pg. 20). No additional formal tests are included. |
| Certainty assessment | 15 | Describe any methods used to assess certainty (or confidence) in the body of evidence for an outcome. | The Methods section describes how GRADE certainty assessment was conducted and was inferred from high DBC quality ratings, low publication bias risk, and meta-regression results (pg. 10). |
| **RESULTS** | | |  |
| Study selection | 16a | Describe the results of the search and selection process, from the number of records identified in the search to the number of studies included in the review, ideally using a flow diagram. | The Results section includes numbers of records identified (2,055), after duplicates (1,687), excluded (1,665), and included (22 studies). Further detail can be found in the Fig 1. PRISMA Flow Chart (pg. 12). |
|  | 16b | Cite studies that might appear to meet the inclusion criteria, but which were excluded, and explain why they were excluded. | There were no studies meeting such criteria. Reasons for exclusion were reported in the Fig 1. PRISMA Flow Chart (pg. 12). |
| Study characteristics | 17 | Cite each included study and present its characteristics. | The Results section (pgs. 12 -18) includes a narrative description of study settings, populations, age, gender, delivery setting, providers, intervention components (Table 2 (intervention characteristics) and Table 3 (study effects) detail this with citations to each included trial. References 64–85 marked as included RCTs. |
| Risk of bias in studies | 18 | Present assessments of risk of bias for each included study. | The Results section includes DBC scores per study in Table 3 (column “DBC”; pg. 18-19). Text summarizing numbers of studies rated excellent, good, fair are included, as well as description of kappa and inter-rater agreement (pg. 20). |
| Results of individual studies | 19 | For all outcomes, present, for each study: (a) summary statistics for each group (where appropriate) and (b) an effect estimate and its precision (e.g. confidence/credible interval), ideally using structured tables or plots. | In the Results section, particularly Table 3, information was provided surrounding study, outcome measure, location, emergency type, population, experimental/control group, T2–T1 difference, and standardized effect size d (pgs. 18-19). Precision (e.g., confidence intervals) for individual study effects are not shown in the excerpt. |
| Results of syntheses | 20a | For each synthesis, briefly summarise the characteristics and risk of bias among contributing studies. | In the Results and Discussion section, there is description of intervention categories (pg. 12), populations (pg. 15-16), settings (pg. 16), and DBC-based quality summary (pg. 20); meta-analysis section provides pooled effect and quality profile (pg. 20). |
|  | 20b | Present results of all statistical syntheses conducted. If meta-analysis was done, present for each the summary estimate and its precision (e.g. confidence/credible interval) and measures of statistical heterogeneity. If comparing groups, describe the direction of the effect. | Meta-analysis section: pooled Hedges’ g = −0.49, 95% CI [−0.82, −0.17], p = 0.0027; τ², I², H², Q described; direction of effect (CBIs reduce PTSD symptoms relative to controls) discussed (pg. 20) |
|  | 20c | Present results of all investigations of possible causes of heterogeneity among study results. | The Results section includes a Moderator Analysis narrative (pg. 22) plus Table 4 (pg. 21-22) showing Hedges’ g, SE, z, p for moderators (intervention type, emergency context, provider, manualization, creative, psychoeducation, cultural adaptation, individual sessions, caregiver sessions). |
|  | 20d | Present results of all sensitivity analyses conducted to assess the robustness of the synthesized results. | Not reported as distinct sensitivity analyses in the text (no analyses such as excluding high risk-of-bias studies or small studies, as none met that criteria, as stated on pg. 9). |
| Reporting biases | 21 | Present assessments of risk of bias due to missing results (arising from reporting biases) for each synthesis assessed. | Results: brief description of funnel plot with moderate asymmetry but high study quality (pg. 20). No formal reporting-bias test results (e.g., Egger) are presented as there were no missing results that impacted analyses (in particular, Barron, Abdallah, & Smith reported effect size, which was used in analysis (rather than calculated from other values that were provided). |
| Certainty of evidence | 22 | Present assessments of certainty (or confidence) in the body of evidence for each outcome assessed. | There is GRADE certainty rating as reported on pg. 10. |
| **DISCUSSION** | | |  |
| Discussion | 23a | Provide a general interpretation of the results in the context of other evidence. | The initial paragraphs of the Discussion summarizes CBIs’ effectiveness and relating findings to prior adult and youth CBI literature, including broader LMIC humanitarian evidence (pgs. 24-27). |
|  | 23b | Discuss any limitations of the evidence included in the review. | The Discussion section provides a strengths and limitations section, with limitations citing heterogeneity, limited follow-up, underrepresentation of subgroups, small samples, attrition, contextual challenges (pgs. 27-29). |
|  | 23c | Discuss any limitations of the review processes used. | The Discussion section provides a strengths and limitations section, with limitations citing notes on subjective judgments in intervention categorization, cultural adaptation coding, limitations of meta-regression and subgroup analyses due to overlapping moderators and heterogeneity (pgs. 27-29). |
|  | 23d | Discuss implications of the results for practice, policy, and future research. | The Conclusion section includes implications for policymakers and implementing agencies, need for investment in CBIs, task-sharing, cultural adaptation, and future research on mechanisms and components (pgs. 29-30). |
| **OTHER INFORMATION** | | |  |
| Registration and protocol | 24a | Provide registration information for the review, including register name and registration number, or state that the review was not registered. | The Methods section includes information on OSF, stating that “the review was registered on the Open Science Framework (OSF). All data, supplementary materials, protocols, and analytic methods are publicly available at: <https://osf.io/wygpv/overview?view_only=5f78fe924bbe4006a0eb0448b626b7a1> (pg. 6). Limitations to registration (i.e., retrospective protocol registration) was mentioned and justified in the limitations section (pg. 28). Prespecified analysis listed. |
|  | 24b | Indicate where the review protocol can be accessed, or state that a protocol was not prepared. | See previous response with OSF link, including all relevant information including protocol (pg. 6). |
|  | 24c | Describe and explain any amendments to information provided at registration or in the protocol. | There were no deviations from or amendments of registered protocol. |
| Support | 25 | Describe sources of financial or non-financial support for the review, and the role of the funders or sponsors in the review. | The Acknowledgements section includes a statement that “this work received no specific funding” (pg. 30). |
| Competing interests | 26 | Declare any competing interests of review authors. | The Acknowledgement section includes a statement that “the authors have declared that no competing interests exist” (pg. 30). |
| Availability of data, code and other materials | 27 | Report which of the following are publicly available and where they can be found: template data collection forms; data extracted from included studies; data used for all analyses; analytic code; any other materials used in the review. | The Methods section, particularly the OSF statement, notes that “All data, supplementary materials, protocols, and analytic methods are publicly available” at OSF link. This includes data extraction materials and analytic code (as stated) (pg. 6). |

*From:*  Page MJ, McKenzie JE, Bossuyt PM, Boutron I, Hoffmann TC, Mulrow CD, et al. The PRISMA 2020 statement: an updated guideline for reporting systematic reviews. BMJ 2021;372:n71. doi: 10.1136/bmj.n71. This work is licensed under CC BY 4.0. To view a copy of this license, visit <https://creativecommons.org/licenses/by/4.0/>
