# Supplementary material for: Effectiveness of community-based interventions for PTSD among youth in low- and middle-income countries affected by humanitarian emergencies: A systematic review and meta-analysis
Source: PLOS Ment Health. 2026 Apr 24;3(4):e0000602. doi: 10.1371/journal.pmen.0000602 (PMC13108866; doi:10.1371/journal.pmen.0000602)
Supplement: S1 Text — Details search strategy, eligibility criteria, data extraction plan, and analysis methods. (DOCX) [file pmen.0000602.s002.docx]

## **1. Title:** Community-based interventions for trauma symptoms in children and adolescents affected by humanitarian emergencies in low- and middle-income countries: A systematic review and meta-analysis protocol.

## **2. Administrative Information**

*Identification:* This document is a protocol for a systematic review and meta-analysis designed to evaluate the effectiveness of community-based interventions (CBIs) in reducing trauma symptoms among youth in low- and middle-income countries (LMICs) affected by humanitarian emergencies. The review will be conducted and reported in accordance with PRISMA 2020 and PRISMA-P guidelines.

*Protocol:* Developed and approved by IP advisor, Dr. M. Claire Greene prior to study initiation – *see amendment 1*

*Author information:*

| Reviewer | Affiliation | Role | Responsibilities |
| --- | --- | --- | --- |
| Gabrielle Drake, MA | Department of Counseling and Clinical Psychology, Teachers College, Columbia University, New York, NY, USA | Project Lead | Overall project oversight, protocol development, study selection, data extraction, statistical analysis, and manuscript preparation. |
| M. Claire Greene, PhD | Program on Forced Migration and Health, Columbia University Mailman School of Public Health, New York, NY, USA | Project Advisor | Provide ongoing supervision across all phases of the project, and oversee the drafting, revision, and submission of the manuscript. |
| – | – | Research Assistant | Participates in data extraction and charting by helping maintain dataset accuracy and consistency through calibration and resolution of coding discrepancies. |

## **3. Introduction**

*Key words:* Community-based interventions, trauma symptoms, PTSD, children, adolescents, youth, low- and middle-income countries, humanitarian emergencies, systematic review, meta-analysis.

*Definitions:*

- Community-Based Interventions (CBIs): Mental health and psychosocial support (MHPSS) interventions implemented outside of traditional healthcare settings, designed to address mental health needs within specific communities. These interventions operate across educational, familial, and social levels, often involving schools, community agencies, and social networks (McLeroy et al., 2003).
- Low- and Middle-Income Countries (LMICs): Countries with a Gross National Income (GNI) per capita between $1,136 and $4,465, as defined by the World Bank Country Lending Group classifications (World Bank, n.d.).
- Humanitarian Emergencies: Disasters or crises (including armed conflict, displacement, and natural disasters) that overwhelm a community's capacity to respond (Boyd et al., 2017).
- Trauma Symptoms: Responses to potentially traumatic events, including symptoms consistent with Post-Traumatic Stress Disorder (PTSD) as defined in the DSM-5, including intrusive symptoms, avoidance, negative changes in cognition and mood, changes in arousal and reactivity, and dissociative symptoms (American Psychiatric Association [APA], 2022).
- Youth: Individuals aged 6–18 years, encompassing children and adolescents across early to late childhood developmental stages.

*Background and Rationale*

Humanitarian emergencies affect millions yearly, with particularly severe consequences in low- and middle-income countries (UNICEF, 2025; Patel et al., 2018). These crises pose substantial threats to children and adolescents, including socioeconomic instability, displacement, family separation, heightened vulnerability to violence, and increased exposure to potentially traumatic events (Betancourt et al., 2013; Reed et al., 2019). The mental health burden following humanitarian emergencies is substantial. In LMICs, the prevalence of PTSD among those exposed to conflict or war is three times higher (15.3%) than in non-affected populations (Charlson et al., 2019). Among young refugees and asylum seekers, 22.7% meet criteria for PTSD, whereas the general lifetime prevalence for PTSD in school age youth is estimated around 2.3% (Fazel et al., 2008; Merikangas et al., 2010). The impacts of untreated trauma are far-reaching, affecting relationships, health, educational outcomes, and long-term psychosocial functioning (Perfect et al., 2016). CBIs offer a practical solution to address treatment gaps in these high-burden, resource-limited contexts. CBIs can accommodate limited resources by leveraging community strengths, engaging local organizations and social networks, and providing flexible, accessible mental health and psychosocial support (Betancourt et al., 2013). Evidence supports the effectiveness of CBIs in reducing symptoms of anxiety, depression, suicidality, and other behavioral disorders among child and adolescent populations in LMICs (Purgato et al., 2018). However, the evidence base for CBIs in addressing youth PTSD symptoms remains heterogeneous, with variable study designs, outcome measures, and intervention components (Stapleton et al., 2020). To date, no comprehensive systematic review has evaluated the effectiveness of CBIs specifically for trauma symptoms in youth in humanitarian-affected LMICs, examined which intervention components are most effective, or explored the mechanisms by which these interventions achieve symptom reduction in high-risk contexts. A systematic review with meta-analysis is warranted to synthesize existing evidence, identify effective intervention characteristics, and clarify implications for clinical practice, policy, and future research.

*Objectives and Review Questions*

The primary objective of this systematic review is to evaluate the effectiveness of community-based interventions in reducing trauma symptoms among youth (ages 6–18 years) in low- and middle-income countries affected by humanitarian emergencies. Secondary, exploratory objectives include identifying intervention characteristics associated with greater effectiveness.

1. What is the overall effectiveness of community-based interventions in reducing trauma symptoms in youth (ages 6–18) in LMICs affected by humanitarian emergencies?
2. Exploratory: Which intervention characteristics are associated with greater effectiveness?
3. Exploratory: Do intervention effects differ by humanitarian emergency context?

## **4. Methods**

*Eligibility criteria*

Population:

- Individuals aged 6–18 years
- Experiencing trauma symptoms or exposed to specified potentially traumatic events
- Residing in or affected by humanitarian emergencies in LMICs

Intervention:

- Community-based mental health interventions implemented outside traditional healthcare settings
  - Focused on reducing trauma symptoms or post-traumatic stress
  - School-based interventions will be included, as schools often function as central community hubs in LMIC contexts

Comparator: control groups, expected to be treatment as usual, enhanced treatment as usual, wait list control, or active control

Outcomes: Trauma symptoms (measured via validated instruments assessing PTSD symptoms or trauma-related distress)

Time: 2010- 2025 (present year)

Setting: Low- and middle-income countries (World Bank classification) affected by humanitarian emergencies.

Design: RCT

Language: English-language publication

*Search terms and strategy:* Searches will be conducted in five academic bibliographic databases to identify all relevant published research:

1. PubMed (searched July 25, 2024)
2. PsycINFO (searched July 25, 2024)
3. Embase (searched July 27, 2024)
4. Cochrane Central Register of Controlled Trials (CENTRAL) (searched August 5, 2024)
5. Scopus (searched August 7, 2024)
   1. Additional Search Methods: Back/forward searching via Google Scholar on key included studies

*Search Strategy, to be documented in PRISMA flow chart:*

- Population: youth OR adolescen* OR child OR teen* OR kid OR young
- Intervention: “mental health” OR psych* OR counseling OR therapy OR program OR intervention OR coaching OR family OR parent* OR prevent* OR promot* OR wellbeing OR wellness OR school-based
- Outcome: trauma OR PTSD OR symptom* OR "post-traumatic stress" OR "post traumatic stress" OR "acute stress" OR "stress disorder" OR "anxiety" OR "depression" OR "psychological distress" OR "emotional distress" OR "emotional regulation" OR "mental distress" OR outcome
- Setting: ("low- and middle-income countries" OR "low- and middle-income country" OR "low and middle income countries" OR "low and middle income country" OR "developing country" OR "developing countries" OR "developing nation" OR "developing nations" OR "developing world" OR "less developed country" OR "less developed countries" OR "less developed nation" OR "less developed nations" OR "middle income country" OR "middle income countries" OR "middle income nation" OR "middle income nations" OR "low income country" OR "low income countries" OR "low income nation" OR "low income nations" OR "lower income country" OR "lower incomecountries" OR lmic OR lmics) AND ("emergency" OR "conflict" OR "disaster")

**Selection Process/Screening**

*Title/abstract and Full-text screening:* The eligibility criteria will be piloted on around 10 records to assess appropriateness eligibility criteria and refine decision rules as needed. Search results will be recorded where the project lead will screen titles and abstracts, recording decisions as Yes, No, or Unclear.

*Handling multiple reports:* Multiple reports of the same study will be identified and linked using study characteristics (e.g., sponsor or ethics number, dates, sample, setting). Linked reports will be merged under a primary reference (most complete and/or most recent), with supplemental reports used to extract any additional data.

**Data Charting and Extraction**

The review team will use a standardized data-charting form using Excel or Google Sheets. This framework will be piloted on a subset of studies and refined as needed, documenting changes made. During full data extraction, two reviewers will independently chart each study, with interrater reliability assessed using Cohen’s kappa. Any discrepancies will be resolved through discussion to reach consensus or deliberation by a third reviewer.

*Anticipated items to be charted - see amendment 2.*

- Publication details: authors, year, country
- Study specific: child age (mean/SD), gender, research question, design, LMIC classification, type of humanitarian context, population specifics (if provided)
- Intervention: program name and description, comparator, study setting, dosage, eligibility, sample size (control and intervention group), PTSD measure, eval points
- Outcomes: intervention effects (quantitative), follow-up effects, outcome summary (qualitative), notes

*Missing data:* If important data are missing or unclear, the team will attempt to contact study authors; unresolved missing items will be coded as such and discussed in limitations.

*Risk of bias:* Two independent reviewers will assess each included RCT using DBC. Disagreements will be resolved through discussion or third-reviewer consultation. Cohen's kappa will be calculated to quantify inter-rater reliability with a target of ≥ 0.60. This will be narratively described or presented in table format.

**Data Synthesis and Presentation of Results**

A random-effects meta-analysis will be conducted to estimate the pooled effectiveness of CBIs on trauma symptoms. Meta-regression and subgroup analyses will explore potential moderators of intervention effectiveness.

Effect Size Calculation (Lin & Aloe, 2021)

- Primary outcome: Change in trauma symptoms from baseline to post-intervention
- Effect size: Hedges' g (bias-corrected standardized mean difference)
  - Calculation: For each study, Hedges’ g will be computed using the change in trauma symptoms from baseline (T1) to post-intervention (T2) in the intervention and control groups (i.e., standardized difference in mean change scores).
- Interpretation: Negative Hedges' g = reduction in trauma symptoms (favorable outcome)

Model: Random-effects meta-analysis (Bakbergenuly, Hoaglin, & Kulinskaya, 2020)

- Outcome: Pooled Hedges' g for trauma symptoms across RCTs
- Random effects: Study-level random intercept, assuming normal distribution of true effects across studies
- Between-study variance (τ²) estimation: Restricted maximum likelihood (REML)
- Output: Pooled estimate with 95% CI, z-test for overall effect, Cochran's Q, I² heterogeneity index, H² statistic
  - Null Hypothesis (H₀): Community-based interventions do not differ from control conditions in their effects on trauma symptoms (pooled Hedges' g = 0).
  - Alternative Hypothesis (H₁, two-tailed): Community-based interventions differ from control conditions in their effects on trauma symptoms (pooled Hedges' g < 0).
  - Moderation Hypothesis: Intervention- and context-specific characteristics (e.g., intervention type, provider, manualization, cultural adaptation, caregiver components, humanitarian emergency context) moderate the effect size, leading to differential trauma symptom reduction across study subgroups.

Heterogeneity (Higgins et al., 2003)

- Cochran's Q-test for statistical heterogeneity (p < 0.10 indicates significant)
- I² statistic (variation due to true between-study differences): <25% low, 25–50% moderate, 50–75% substantial, >75% considerable
- Visual inspection of forest plots

Moderator Analyses: Meta-Regression – Categorical moderators will be examined via separate mixed-effects meta-regression models to identify intervention and study features associated with differential effectiveness:

- Moderators to be exploratory, and as such anticipate to be added after screening once more information about study characteristics are garnered

Potential Moderators (tested in separate models) – se*e amendment 3*

1. Intervention Type/Categorization
2. Provider Type: Professional vs. paraprofessional/community
3. Manualization: Manualized vs. non-manualized
4. Cultural Adaptation: Adapted vs. standard protocol
5. Humanitarian Emergency Context

- Moderator: One categorical predictor per model
- Omnibus Test: Cochran's Q-test for moderators with p-value and R²
- Subgroup Effect Estimates: Model intercept represents reference group; coefficients represent differences from reference

Software and Code: All meta-analyses will be conducted using R software

Sensitivity: To assess robustness of the estimate (Cochrane, n.d.) *–see amendment 4*

1. Exclusion of studies with high risk of bias (DBC)
2. Exclusion of outlier studies with extreme effect sizes
3. Restriction to studies with low attrition (<20%)
   1. Assessed through funnel plot and bias measurements (DBC)

*Certainty*: Assessed qualitatively based on the GRADE approach domains

- Risk of Bias: DBC assessments (see previous section)
- Precision: I², heterogeneity measures, and width of pooled 95% CI
- Directness: Relevance of studies to population and outcomes of interest
- Publication Bias: Funnel plot
- Magnitude of Effect: Size of pooled Hedges' g and subgroup estimates
  - Qualitatively summarized (e.g., high, moderate, low) based on these domains

*Ethics:* This is a systematic review of published studies. No original human subject’s data will be collected. Formal ethics approval is not required. All data analyzed will be publicly available upon request – s*ee amendment 5*

*Dissemination:* Submission as IP project for Teachers College, Columbia University in partial fulfillment of master’s degree requirements. - *see amendment 6*

*Funding Sources/Sponsors:* N/A

*Conflicts of Interest:* The authors have no conflicts of interest to declare

*Acknowledgements:* The team will acknowledge any non-author contributors in the final manuscript.

*Amendments:* Any amendments to this protocol will be documented with a description of the change, the rationale, and the stage of the amendment.

**References**

American Psychiatric Association. (2022). *Diagnostic and statistical manual of mental disorders* (DSM-5-TR). American Psychiatric Association Publishing. <https://doi.org/10.1176/appi.books.9780890425787>

Bakbergenuly, I., Hoaglin, D. C., & Kulinskaya, E. (2020). Estimation in meta-analyses of mean difference and standardized mean difference. *Statistics in medicine*, *39*(2), 171–191. https://doi.org/10.1002/sim.8422

Betancourt, T. S., Newnham, E., Layton, H., Kim, H. G., Steinberg, A. M., Ellis, H., & Birman, D. (2013). Trauma history and psychopathology in war-affected refugee and asylum seeking children referred to a child and adolescent mental health service. *European Child & Adolescent Psychiatry, 22*(11), 691–700. <https://doi.org/10.1007/s00787-013-0412-5>

Boyd, A. T., Cookson, S. T., Anderson, M., Bilukha, O. O., Brennan, M., Handzel, T., Hardy, C., Husain, F., Cardozo, B. L., Colorado, C. N., Shahpar, C., Talley, L., Toole, M., & Gerber, M. (2017). Centers for Disease Control and Prevention Public Health Response to Humanitarian Emergencies, 2007-2016. *Emerging infectious diseases*, *23*(13), S196–S202. https://doi.org/10.3201/eid2313.170473

Bullers, K., Howard, A. M., Hanson, A., Kearns, W. D., Orriola, J. J., Polo, R. L., & Sakmar, K. A. (2018). It takes longer than you think: Librarian time spent on systematic review tasks. *Journal of the Medical Library Association*, *106*(2), 198–207. https://doi.org/10.5195/jmla.2018.323

*Campbell Library*. (n.d.). Retrieved <https://campbellcollaboration.org/library/campbell-systematic-review-templates.html>

*Charlson, F., van Ommeren, M., Flaxman, A., Cornett, J., Whiteford, H., & Saxena, S. (2019). New WHO prevalence estimates of mental disorders in conflict settings: A systematic review and meta-analysis. The Lancet, 394(10194), 240–248.* [*https://doi.org/10.1016/S0140-6736(19)30934-1*](https://doi.org/10.1016/S0140-6736(19)30934-1)

*Chapter 7: Considering bias and conflicts of interest among the included studies | Cochrane*. (n.d.). https://www.cochrane.org/authors/handbooks-and-manuals/handbook/current/chapter-07

Covidence, 2025; A practical guide: Protocol Development for Systematic Reviews, Covidence

*Evidence Synthesis Service*. (n.d.). Cornell University Library. Retrieved https://www.library.cornell.edu/services/evidence-synthesis/how-librarians-can-help

Fazel, M., Wheeler, J., & Danesh, J. (2008). Prevalence of serious mental disorder in 7000 refugees resettled in western countries: A systematic review. *The Lancet, 372*(9651), 1309–1314. <https://doi.org/10.1016/S0140-6736(08)61659-9>

Grasso, D. J., Ford, J. D., & Briggs-Gowan, M. J. (2013). Early life trauma exposure and stress sensitivity in young children. *Journal of Pediatric Psychology*, *38*(1), 94–103. <https://doi.org/10.1093/jpepsy/jss101>

Guyatt, G. H., Oxman, A. D., Vist, G. E., Kunz, R., Falck-Ytter, Y., Alonso-Coello, P., & Schünemann, H. J. (2008). GRADE: An emerging consensus on rating quality of evidence and strength of recommendations. BMJ, 336(7650), 924–926. https://doi.org/10.1136/bmj.39489.470347.AD

Heleniak, C., Jenness, J. L., Vander Stoep, A., McCauley, E., & McLaughlin, K. A. (2016). Childhood maltreatment exposure and disruptions in emotion regulation: A transdiagnostic pathway to adolescent internalizing and externalizing psychopathology. *Cognitive Therapy and Research*, *40*(3), 394–415. <https://doi.org/10.1007/s10608-015-9735-z>

Higgins, J. P. T., Thompson, S. G., Deeks, J. J., & Altman, D. G. (2003). Measuring inconsistency in meta-analyses. *BMJ*, *327*(7414), 557–560. <https://doi.org/10.1136/bmj.327.7414.557>

Kane, J. C., Ventevogel, P., Spiegel, P., Bass, J. K., van Ommeren, M., & Bolton, P. A. (2016). Mental, neurological, and substance use problems among refugees in primary health care: Analysis of pan-European refugee health records. *BMC Public Health, 16*(1), 438. <https://doi.org/10.1186/s12889-016-3038-0>

Lin, L., & Aloe, A. M. (2021). Evaluation of various estimators for standardized mean difference in meta-analysis. *Statistics in medicine*, *40*(2), 403–426. https://doi.org/10.1002/sim.8781

Merikangas, K. R., He, J. P., Burstein, M., Swanson, S. A., Avenevoli, S., Cui, L., Benjet, C., Georgiades, K., & Swendsen, J. (2010). Lifetime prevalence of mental disorders in U.S. adolescents: Results from the National Comorbidity Survey Replication–Adolescent Supplement (NCS-A). *Journal of the American Academy of Child & Adolescent Psychiatry, 49*(10), 980–989. <https://doi.org/10.1016/j.jaac.2010.05.017>

McLeroy, K. R., Norton, B. L., Kegler, M. C., Burdine, J. N., & Sumaya, C. V. (2003). Community-based interventions. *American Journal of Public Health*, *93*(4), 529–533. <https://doi.org/10.2105/AJPH.93.4.529>

Patel, V., Saxena, S., Lund, C., Thornicroft, G., Baingana, F., Bolton, P., Chisholm, D., Collins, P. Y., Cooper, J. L., Eaton, J., Herrman, H., Herzallah, M. M., Huang, Y., Jordans, M. J. D., Kleinman, A., Medina-Mora, M. E., Morgan, E., Niaz, U., Omigbodun, O., … UnÜtzer, J. (2018). The Lancet Commission on global mental health and sustainable development. *The Lancet, 392*(10157), 1553–1598. <https://doi.org/10.1016/S0140-6736(18)31612-X>

Perfect, M. M., Turley, M. R., Carlson, J. S., Yohanna, J., & Saint Gilles, M. P. (2016). School-related outcomes of traumatic event exposure and subjective stress: A multimethod assessment. *Journal of School Psychology, 55*, 1–14. <https://doi.org/10.1016/j.jsp.2015.12.001>

*PRISMA-P-checklist*. (n.d.). Retrieved <http://prisma-statement.org/documents/PRISMA-P-checklist.pdf>

Purgato, M., Gastaldon, C., Papola, D., van Ommeren, M., Barbui, C., & Tol, W. A. (2018). Psychological therapies for the treatment of mental disorders in low- and middle-income countries: A meta-analysis and systematic review. *Epidemiology and Psychiatric Sciences, 27*(2), 115–127. <https://doi.org/10.1017/S2045796017000330>

Reed, R. V., Fazel, M., Jones, L., Panter-Brick, C., & Stein, A. (2019). Mental health of displaced and refugee children resettled in low-income and middle-income countries: Risk and protective factors. *The Lancet, 394*(10210), 180–190. <https://doi.org/10.1016/S0140-6736(19)31550-9>

Shamseer, L., Moher, D., Clarke, M., Ghersi, D., Liberati, A., Petticrew, M., Shekelle, P., Stewart, L. A., & the PRISMA-P Group. (2015). Preferred reporting items for systematic review and meta-analysis protocols (Prisma-p) 2015: Elaboration and explanation. *BMJ*, *349*(jan02 1), g7647–g7647. <https://doi.org/10.1136/bmj.g7647>

Stapleton, L., Garamvolgyi, M., & Miller, K. E. (2020). Task sharing for the delivery of psychosocial interventions in humanitarian settings: A systematic review. *Global Mental Health, 7*, e27. <https://doi.org/10.1017/gmh.2020.21>

*Systematic and scoping reviews: Before you start*. (n.d.). Western University Libraries.

UNICEF. (2025). *UNICEF humanitarian action for children 2025 overview*. <https://www.unicefusa.org/sites/default/files/2024-12/UNICEF-Humanitarian-Action-for-Children-2025-Overview.pdf>

World bank country and lending groups – world bank data help desk. (n.d.). https://datahelpdesk.worldbank.org/knowledgebase/articles/906519-world-bank-country-and-lending-groups
