# Supplementary material for: Effectiveness of community-based interventions for PTSD among youth in low- and middle-income countries affected by humanitarian emergencies: A systematic review and meta-analysis
Source: PLOS Ment Health. 2026 Apr 24;3(4):e0000602. doi: 10.1371/journal.pmen.0000602 (PMC13108866; doi:10.1371/journal.pmen.0000602)
Supplement: S1 Table — Documents deviations between the original master’s thesis plan and actual conducted review, with rationale, timing, and impact assessments. (DOCX) [file pmen.0000602.s003.docx]

## **Protocol Amendments**

| Amendment # | Original Protocol Plan | Actual Conducted | Date of Change | Rationale | Impact on Results |
| --- | --- | --- | --- | --- | --- |
| 1 | No formal registration planned (master's thesis) | Retrospective OSF registration | Manuscript preparation | Registration was required for journal submission and aligns with best practices in evidence synthesis | None. The protocol was developed and approved prior to study initiation by the supervising advisor, minimizing risk of bias |
| 2 | Extraction of a focused set of study- and intervention-level variables | Expanded and operationalized data extraction framework with additional structured fields (e.g., intervention provider, manualization, cultural adaptation, caregiver involvement, session type, and setting characteristics) | During pilot data extraction | To improve consistency, reduce ambiguity, and enable systematic coding of intervention characteristics for moderator analyses | No impact on primary outcomes or effect size estimation; facilitated more precise and reproducible moderator analyses |
| 3 | Moderator analyses using a limited set of broad categories | Expanded moderator framework including intervention type, humanitarian context, provider type, manualization, cultural adaptation, psychoeducation, creative components, and session structure | During Analysis Phase | To more precisely operationalize intervention characteristics and test theoretically relevant sources of heterogeneity identified in the review framework | No impact on primary pooled estimates; enhanced interpretability of heterogeneity and subgroup findings |
| 4 | Data available upon request | Public sharing of protocol, data extraction files, and analytic code via OSF | Manuscript preparation | To enhance transparency, reproducibility, and alignment with open science practices | No impact on results; improves accessibility and transparency |
| 5 | Submission as master’s thesis only | Submission as thesis and preparation for peer-reviewed publication | Manuscript preparation | To disseminate findings more broadly and contribute to the evidence base | No impact on methods or findings |
| 7 | Standard meta-analytic models only | Additional post hoc diagnostic and sensitivity analyses, including leave-one-out analyses, influence diagnostics (e.g., Cook’s distance, DFFITS, studentized residuals), and cluster-adjusted models using varying ICC assumptions | After primary meta-analysis | To evaluate the robustness of findings and assess the influence of individual studies and potential clustering effects identified following initial model estimation | No change to the direction or significance of primary findings; results were robust across diagnostic checks. These analyses strengthen confidence in the stability of the reported effects |
