# Supplementary material for: Effectiveness of community-based interventions for PTSD among youth in low- and middle-income countries affected by humanitarian emergencies: A systematic review and meta-analysis
Source: PLOS Ment Health. 2026 Apr 24;3(4):e0000602. doi: 10.1371/journal.pmen.0000602 (PMC13108866; doi:10.1371/journal.pmen.0000602)
Supplement: S2 Table — For the primary outcome comparing community-based interventions to control conditions. (DOCX) [file pmen.0000602.s004.docx]

Summary of Findings Table – GRADE Approach to Assess Certainty of Evidence

| **Outcome** | **Comparison** | **Participants and Studies** | **Effect** | **Certainty of the evidence (GRADE)** | **Plain language summary** | **Reasons for rating** |
| --- | --- | --- | --- | --- | --- | --- |
| Post‑intervention trauma symptoms (continuous; standardized mean difference across validated scales) | Community‑based intervention vs control (treatment as usual, enhanced TAU, waitlist, or active comparison) | N = 3,665 participants (21 RCTs) | Hedges’ g = −0.5 (95% CI −0.84 to −0.17) | ⊕⊕⊕◯ Moderate | Community‑based interventions probably reduce trauma symptoms compared to control conditions. | Certainty started at high (all RCTs) and was downgraded one level for inconsistency due to very high heterogeneity (I² = 96.42%; Q(20) = 170.0984, p < 0.001). It was not downgraded for risk of bias (good methodological quality based on DBC ratings), indirectness (populations, interventions, and outcomes closely match the review question), imprecision (pooled effect is clearly different from no effect with a reasonably narrow CI), or publication bias (funnel plot and regression test for asymmetry did not suggest substantial small‑study effects). |

Explanations: The available evidence comes from 21 randomized controlled trials including 3,665 children and adolescents (ages 6–18) exposed to conflict, displacement, or natural disasters in low- and middle-income countries. Community-based psychological interventions are compared against control conditions, including treatment as usual, enhanced treatment as usual, waitlist, or other active controls. The primary outcome is post-intervention trauma/PTSD symptom severity, measured with validated child PTSD instruments (e.g., CRIES 8, CRIES 13, CPSS, UCLA PTSD RI), with no additional outcomes formally graded. In control groups, typical post-intervention PTSD symptom levels remained in the mild-to-moderate range on these scales, providing a baseline against which improvements in intervention groups were judged. The pooled relative effect indicated that community-based interventions probably reduce trauma symptoms compared with control (Hedges’ g = −0.5, 95% CI −0.84 to −0.17), representing a moderate standardized reduction in symptoms. Across the 21 RCTs (N = 3,665; 2,330 intervention, 1,345 control), the GRADE assessment for this primary outcome rated the certainty of evidence as moderate, with one level downgraded for inconsistency due to heterogeneity and no further downgrades for risk of bias, indirectness, imprecision, or publication bias. We note the high heterogeneity, the reliance on standardized mean differences across different PTSD measures, and the contextual factors that may influence generalizability.

**References**

*Chapter 14: Completing ‘Summary of findings’ tables and grading the certainty of the evidence | Cochrane*. (n.d.). Retrieved from <https://www.cochrane.org/hr/authors/handbooks-and-manuals/handbook/current/chapter-14?utm_source=chatgpt.com#section-14-1>

Santesso, N., Carrasco-Labra, A., Langendam, M., Brignardello-Petersen, R., Mustafa, R. A., Heus, P., Lasserson, T., Opiyo, N., Kunnamo, I., Sinclair, D., Garner, P., Treweek, S., Tovey, D., Akl, E. A., Tugwell, P., Brozek, J. L., Guyatt, G., & Schünemann, H. J. (2016). Improving GRADE evidence tables part 3: Detailed guidance for explanatory footnotes supports creating and understanding GRADE certainty in the evidence judgments. *Journal of Clinical Epidemiology*, *74*, 28–39. <https://doi.org/10.1016/j.jclinepi.2015.12.006>

The Cochrane Collaboration. (n.d.). *GRADEpro GDT*. Cochrane Methods. Retrieved from [https://methods.cochrane.org/gradeing/gradepro-gdt](https://methods.cochrane.org/gradeing/gradepro-gdt?utm_source=chatgpt.com)
