# Supplementary figures and images for: Effectiveness of community-based interventions for PTSD among youth in low- and middle-income countries affected by humanitarian emergencies: A systematic review and meta-analysis
Source: PLOS Ment Health. 2026 Apr 24;3(4):e0000602. doi: 10.1371/journal.pmen.0000602 (PMC13108866; doi:10.1371/journal.pmen.0000602)

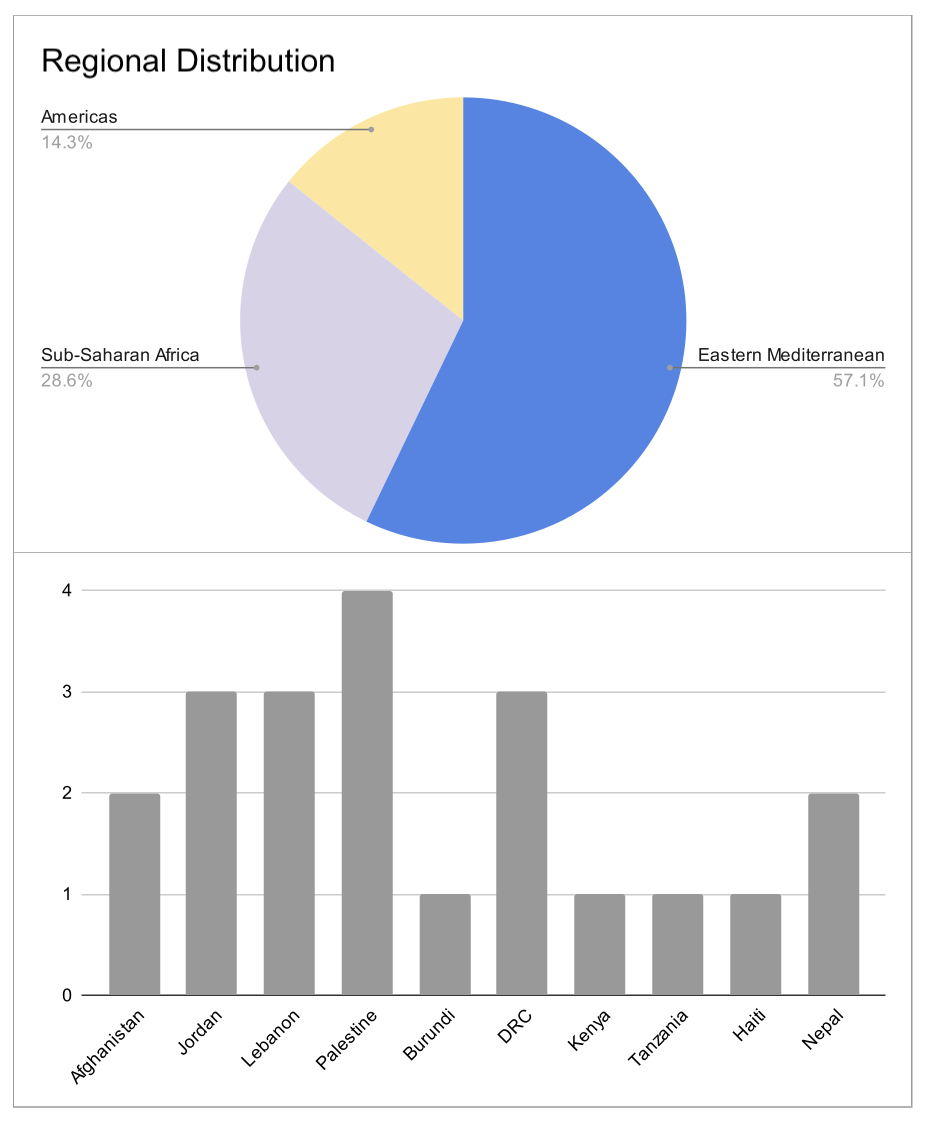

Supplement: S1 Fig — Pie and bar chart showing distribution of studies by geographic region. (TIFF) [file pmen.0000602.s005.tiff]
